# The time-resolved transcriptome of *C. elegans*

**DOI:** 10.1101/gr.202663.115

**Published:** 2016-10

**Authors:** Max E. Boeck, Chau Huynh, Lou Gevirtzman, Owen A. Thompson, Guilin Wang, Dionna M. Kasper, Valerie Reinke, LaDeana W. Hillier, Robert H. Waterston

**Affiliations:** 1 Department of Genome Sciences, School of Medicine, University of Washington, Seattle, Washington 98195, USA;; 2 Department of Biology, Regis University, Denver, Colorado 80221, USA;; 3 Department of Genetics, School of Medicine, Yale University, New Haven, Connecticut 06520, USA

## Abstract

We generated detailed RNA-seq data for the nematode *Caenorhabditis elegans* with high temporal resolution in the embryo as well as representative samples from post-embryonic stages across the life cycle. The data reveal that early and late embryogenesis is accompanied by large numbers of genes changing expression, whereas fewer genes are changing in mid-embryogenesis. This lull in genes changing expression correlates with a period during which histone mRNAs produce almost 40% of the RNA-seq reads. We find evidence for many more splice junctions than are annotated in WormBase, with many of these suggesting alternative splice forms, often with differential usage over the life cycle. We annotated internal promoter usage in operons using SL1 and SL2 data. We also uncovered correlated transcriptional programs that span >80 kb. These data provide detailed annotation of the *C. elegans* transcriptome.

RNA transcripts represent a direct readout of the information stored in a genome. Their differential abundance in turn reflects the regulatory networks operative in the organism. Accurate and comprehensive characterization of RNA transcript levels is central to an understanding of how an organism's genome dictates its traits and behavior. In the nematode *Caenorhabditis elegans*, multiple different studies assayed the RNA content at different stages and in different tissues. Microarray studies, including a detailed embryonic time course using small numbers of hand-picked embryos, gave a picture of overall gene expression across early development (Kim et al. 2001; Baugh et al. 2003; Levin et al. 2012). SAGE tags provided a deeper analysis of transcripts present at various stages and in certain tissues or cell types (Shin et al. 2008; McGhee et al. 2009). More recently, CEL-seq has been used on individual embryos to produce a detailed embryonic time series (Hashimshony et al. 2015).

Each of the aforementioned studies has provided useful insight into the RNA transcripts present during the life cycle, but none cover the entire life cycle and each has its own shortcomings. Microarray studies have a limited dynamic range, often assay only annotated genes, fail to distinguish between close paralogs, and usually ignore different isoforms. Studies using small numbers of embryos require multiple rounds of amplification, possibly introducing significant distortion into the expression measurements. SAGE tags attempt only to assay 3′ ends of polyadenylated [poly(A)] transcripts, ignoring splicing; internal priming at A-rich sites can create false positive tags. In addition, the short length of early SAGE tags led to ambiguity in genome alignment. CEL-seq on individual embryos can assay very precise time points, but again the method only seeks to count 3′ ends of poly(A) mRNAs. In addition, the limited efficiency of CEL-seq in copying RNA into DNA and the subsequent amplification leads to irregular representation of lower abundance transcripts. The lack of a single comprehensive data set across the full life cycle complicates comparison of gene expression levels at different stages.

To provide a comprehensive, high quality, uniformly collected expression data set for *C. elegans*, we performed RNA-seq on bulk samples from synchronized animals across the full life cycle, including embryonic samples beginning at four cells and sampled at 30-min intervals. Obtaining these embryo data required the development of a novel method to synchronize bulk populations of embryos and the implementation of a Bayesian approach to refine the estimates of gene expression within individual developmental series and to combine multiple series. The resultant new embryo data, combined with expression data from larval stages, dauers, males, and aged adults collected as part of the modENCODE project (Hillier et al. 2009; Gerstein et al. 2010, 2014), reveal the pattern of expression for protein coding genes, as well as the patterns of noncoding transcripts, splice junctions, and spliced leader sequences across the full life cycle.

## Results

### Data sets

In earlier papers, we reported quantitative analyses of gene expression data based on RNA-seq analysis for mid-larval stages, young adults with oocytes but no embryos, dauers, and animals entering and exiting the dauer stage as well as L4 males (see Methods for a detailed description of the post-embryonic stages sampled) (Hillier et al. 2009; Gerstein et al. 2010, 2014). To obtain a higher resolution description of the changes of gene expression in embryogenesis, and to thus provide greater insight into shared and distinct expression patterns and their underlying gene regulatory networks, we developed a method to obtain large numbers of embryos in which the bulk of the population in the starting sample was within a 1-h developmental window (see Methods for details). Four independent time series were collected, one selecting for poly(A) containing mRNAs (Gerstein et al. 2014) and three using rRNA subtraction on total RNA samples so as to include mRNAs lacking poly(A), yielding a total of 63 samples with high coverage, high quality data (Supplemental Table S1). With the previously reported data for other life stages (Gerstein et al. 2014), we report here the generation of almost 2.5 billion mapped reads across 93 different samples.

To refine the estimates of expression per stage and to combine the different developmental series more effectively, we developed a Bayesian approach that exploits the expression data itself to estimate the stages present in each sample and the expression of each gene at every stage (see Methods for details) (Francesconi and Lehner 2014). Through this method, we were able to compensate for slight differences in the composition of the starting populations and differences between series in growth rates attributable to minor differences in temperature, growth conditions, or other factors (Supplemental Fig. S1). We estimated the composition and the average time of each experimental sample on a common relative scale. To convert this relative scale to embryonic time, we mapped each sample to the most complete series (0223) collected from a single, relatively coherent starting population grown at 20°C (Supplemental Fig. S2). We confirmed the assignment by comparing the results to those recently published using single embryo analysis (Supplemental Fig. S3; Hashimshony et al. 2015). We used these embryo results along with weighted averages of the replicate data sets for each of the larval and adult stages, the dauer stages (entry, dauer, and exit), and the L4 males to measure transcript expression throughout the life cycle of *C. elegans*. To complement these data sets, we added single samples of hand-picked four-cell embryos, dissected adult hermaphrodite gonads, and older adults lacking sperm (*spe-9* mutants). The four-cell sample provides a highly synchronized population just as zygotic transcription is beginning for comparison with the bulk embryonic series, and the latter two samples provide information about the origin of transcripts in the early embryo. The sequence data are available at the Sequence Read Archive (see Data access).

### Protein coding gene expression patterns

We began our analysis of the RNA-seq data by looking at the relative expression levels of protein coding genes across all of the stages (Supplemental Table S2). The number of genes expressed above threshold rises in early embryogenesis and again in late embryogenesis, falls in the first larval stage, and rises yet again in young adults. L4 males exhibit the highest number of genes above threshold (Supplemental Fig. S4).

**Figure 1.**
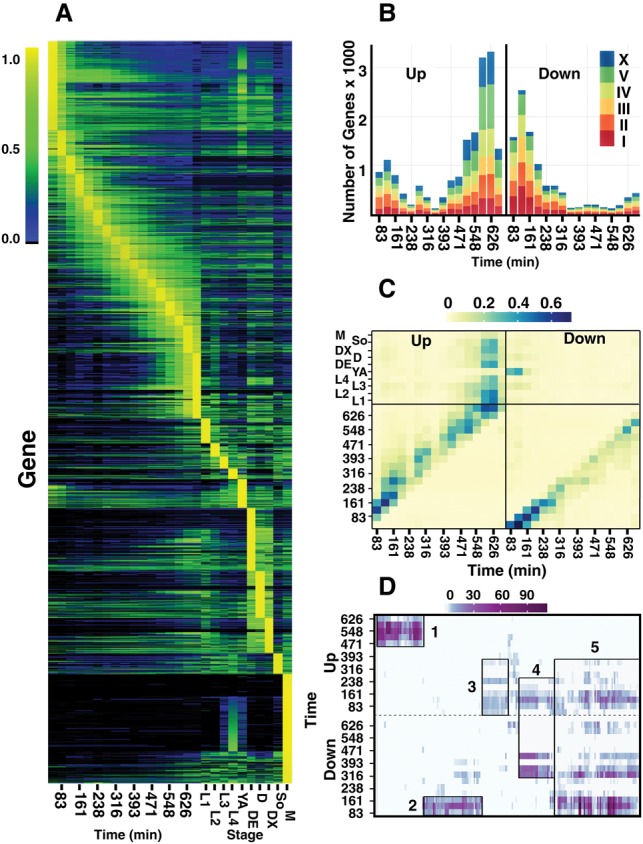
Gene expression dynamics across all stages. (*A*) Normalized expression across embryogenesis and post-embryonic time points clustered by the stage of maximal expression (see Methods for details). Normalized expression is colored from none (black), to low (blue), to medium (green), and to maximal (yellow) with the scale provided on the *left* running from 0% to 100% of maximal expression per gene. Only genes (17,401) with at least one stage with expression >0.07 dcpm (depth of coverage per base per million reads) are shown. Embryonic stages on the *left* half of the plot are given in minutes, post-two-cell embryo. Post-embryonic stages include the four larval stages (L1–L4), young adult (YA), dauer entry (DE), dauer (D), dauer exit (DX), adult soma (SO), and L4 stage males (M). (*B*) Genes up-regulated (*left*) and down-regulated (*right*) in one stage relative to the previous time point are shown for each time point in embryogenesis. Gene counts for Chromosomes I, II, III, IV, V, and X are colored red, dark orange, light orange, yellow, green, and blue, respectively. (*C*) The proportion of genes overlapping between maximal expression clusters (*y*-axis) and those genes called as up-regulated (*left*) or down-regulated (*right*; *x*-axis) is shown for each embryonic stage. The proportion is colored from light yellow (0) to dark blue (0.65). (*D*) GO term enrichments for each up-regulated (*top*) and down-regulated (*bottom*) set of genes for each time point are clustered and then plotted against the embryonic time points. The significance of the enrichment at a particular time point (negative log of the *P*-value) is given from zero (white) to dark purple (*P* = 10^−110^). Five larger clusters of GO terms are highlighted in rectangles. For example, the fifth cluster has a down, up, down pattern.

To learn more about the patterns of expression of individual genes, we calculated the relative expression of each gene across all stages, and plotted the results, ordering genes by their maximal expression per stage (graphical representations of the gene expression data for each gene across the life cycle are available at GExplore; http://genome.sfu.ca/gexplore) (Fig. 1A). The L4 male sample has the largest number of genes with maximal expression in that stage (2554) as well as the largest number of genes expressed above threshold in any of the stages (15,910) but one (Supplemental Fig. S4). Notably, expression of some genes appears specific for the male, and many others have appreciable expression principally in L4 hermaphrodite larvae, a stage which both shares common L4-specific genes and makes sperm. The first embryonic stage also has a large number of genes with maximal expression (2082), with many genes showing relatively high levels of expression in adjacent stages and also the young adult sample, suggesting that many of these mRNAs are maternally derived and rapidly degraded. The different dauer larvae samples also show high numbers of genes maximally expressed (1476 in dauer entry, 1101 in dauer, and 819 in dauer exit) with relatively high expression of many of these genes in other dauer stages, emphasizing the unusual nature of the dauer state, a developmental variant that arrests in response to environmental stress. The high numbers of genes maximally expressed in the last stage of embryogenesis (1536) presumably reflect the terminal differentiation of many specialized tissues occurring as the animal prepares to hatch.

The heatmap shown in Figure 1A suggests that although many genes are changing in expression both early and late in embryogenesis, many fewer are changing mid-embryogenesis. We looked at this more directly, using the credible intervals produced by the Bayesian unification model to look for genes up- or down-regulated between adjacent stages of embryogenesis (Fig. 1B). In agreement with an earlier report (Levin et al. 2012), the data suggest three periods of large-scale gene regulation. The first period shows large numbers of genes down-regulated and a smaller but still substantial number of genes up-regulated. These changes likely reflect the degradation of maternally derived mRNAs and the onset of zygotic transcription. The second period is relatively quiescent with fewer genes showing change. The last period is dominated by a large number of up-regulated genes that corresponds temporally with the cessation of cell division and the onset of terminal differentiation. More than 10% of all genes show up-regulation in this period. We also looked at up- and down-regulated genes using change-point analysis (Green 1995) and with edgeR (Robinson et al. 2010) and found similar trends (Supplemental Fig. S5; Supplemental Tables S3, S4). The up- and down-regulated genes in each stage appear to be similarly distributed across the five autosomes and the sex chromosome, with the exception that the early down-regulated genes are underrepresented on the X, consistent with a paucity of maternally expressed genes on the X. Looking at specific gene classes (Supplemental Fig. S6), we noted, for example, that the *tbx* class of transcription factors, a group defined by the gene name and known for their role in embryonic development, were either maternally inherited or went up in the first stages and then fell quickly in later stages. In contrast, the *ceh* group of homeodomain transcription factors rose more broadly in embryogenesis with many falling in the transition from late embryo to L1 larva. The F-box genes involved in ubiquitination were up-regulated early in embryogenesis, falling rapidly thereafter. This pattern of expression suggests these F-box genes may be involved in the degradation of maternal proteins or perhaps proteins produced by maternal RNAs.

We looked to see if these up- and down-regulated genes were expressed at other stages in the life cycle (Fig. 1C). As expected, many of the down-regulated genes in the first two stages have maximal expression either in the first embryonic stage or in the young adult and not in other larval stages, consistent with a maternal origin for these RNAs. Genes up-regulated late in embryogenesis show maximal expression in early larval stages and, intriguingly, in the dauer samples.

To learn more about the biological processes associated with these patterns of gene expression, we examined GO terms that were enriched in each up- and down-regulated gene set (Supplemental Fig. S7; Supplemental Tables S5, S6). Clustering the GO terms produced five main clusters (Fig. 1D). The first cluster is heavily enriched in genes up-regulated late in embryogenesis. Representative terms from this cluster include G-coupled protein, ion transport, and synapse development, all terms associated with the development of neurons and the concomitant ability to sense the environment. The second cluster is enriched for genes down-regulated early in development. Representative terms from this cluster include cell cycle progression, endocytosis, and P-granules terms. The third cluster is enriched for genes up-regulated early in development. Almost all of these terms are associated with gene regulation and transcription, consistent with specification of cell fates during this period. The fourth and fifth clusters of GO terms are associated with genes that are both up- and down-regulated over time. The fourth cluster is up-regulated early and then down-regulated during the middle of development and is enriched for terms associated with chromosomal organization, conformational change, and chromatin assembly. The predominance of these terms is consistent with a transition in chromatin from the pluripotent state of the fertilized egg to a more differentiated state (Yuzyuk et al. 2009). It may also reflect the high rate of cell division in the first half of embryogenesis (see “Histones”). The fifth cluster has a down, up, down pattern and is enriched for various metabolic functions and developmental processes that become more or less important as cell types develop.

### Gene expression order

The fate of several tissues in *C. elegans* is determined early in embryogenesis by an ordered series of transcription factor activation. To determine whether the time series data was of sufficient resolution and sensitivity to detect these events occurring in just a fraction of the cells, we used the change-point analysis on the unified embryonic data set to determine the peak of expression of all genes as well as the rate of change across the time course for a maximum of four intervals. The resultant data readily detected the *med-1–end-3–end-1–elt-7–elt-2* cascade that specifies the intestinal fate as well as the *tbx-35/pal-1–(hnd-1/hlh-1)–unc-120* cascade involved in muscle specification (Supplemental Table S7; Baugh et al. 2003; Broitman-Maduro et al. 2009; Raj et al. 2010; Krause and Liu 2012). Thus, the data have adequate time resolution and sensitivity to order these critical events. With the caveat that the data is derived from whole animals, this ordering of gene expression can be used to refine the possible regulatory relationships between the various transcription factors among themselves as well as their targets.

### Splice junctions

In addition to examining the overall expression of the protein coding genes, we also used the RNA-seq data to find splice junctions and to determine the differential usage of alternative splice junctions (introns) over the life cycle (Supplemental Tables S8, S9). A total of 208,627 splice junctions met our false discovery rate (FDR) thresholds (0.05) in at least one sample. However, false positives can accumulate across the many samples that were assayed. To reduce the number of potential false positives, we demanded that a junction be observed in more than one sample and appear at 1% of the level of the average of other junctions within the gene (a 1% threshold for a broadly expressed gene should allow splice junctions used in just 5–10 cells to be detected) (Hillier et al. 2009). This filter reduced the total to 171,827 junctions. The 171,827 junctions confirm most WormBase annotated junctions (110,099/117,223), and most of the WormBase junctions unrepresented in our data sets are listed as “annotated” but not “confirmed” in WormBase (Supplemental Fig. S8). Although most junctions are detected in multiple different stages, more than 6800 are found only in a particular stage; of these, a striking 2419 are found only in the L4 male samples (Supplemental Figs. S9, S10). This large fraction may reflect the genes only or predominantly expressed in males, e.g., 56 of 265 c-type lectin domain (*clec*) genes, but also could include alternative junctions used only in males. In addition to the junctions overlapping WormBase coding genes, our set contains 61,728 junctions not annotated in WormBase. These junctions include 6728 that appear to extend WormBase gene models, many of which appear by the length of the intron to provide alternative 5′ ends of current genes. Another 17,242 lie entirely outside WormBase transcript models. The bulk of the latter set can be joined to WormBase models through a series of exons and splice junctions thus further extending WormBase models, but 6642 appear to be unrelated to any annotated transcript. Most of these latter splice junctions are only weakly expressed.

Many of these junctions share donor or acceptor sites (84,902), making them sites for alternative splicing. The general types of alternative splice forms and their frequencies in *C. elegans* have been described (Gerstein et al. 2014). Here, we first compared the representation of these junctions with constitutive junctions, i.e., those that do not share sites with other junctions and do not overlap exons (70,075). This analysis revealed a bimodal distribution for the sites with multiple junctions, where one portion resembles the distribution of constitutive junctions, but the second peak has much lower representation (Supplemental Fig. S11). This bimodal distribution is consistent with the notion that at alternatively spliced sites, one junction is the major form, and the other junction represents the minor or alternative form.

**Figure 2.**
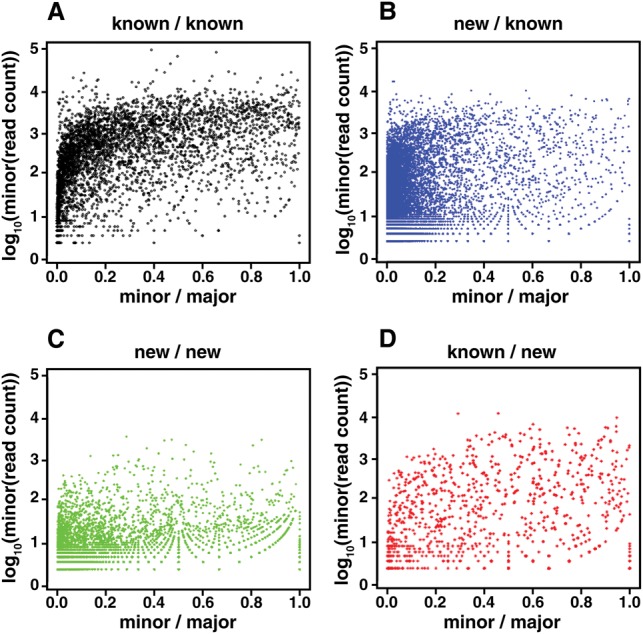
The relative abundance of novel versus known junctions in alternatively spliced pairs within WormBase gene models. For each splice junction site that could be spliced to two or more other sites, i.e., alternatively spliced, we calculated the ratio of reads for each minor form versus the major form and the numbers of reads spanning the minor form. (*A*) Sites where both the major and minor isoforms were both present in WormBase. (*B*) Sites where the major isoform was present in WormBase but the minor form was not. (*C*) Sites where neither the major nor minor isoform were present in WormBase. (*D*) Sites where the minor form was present in WormBase, but not the major isoform. For the case in which the minor form is novel and the major form is known (*B*), a larger fraction of the minor form junctions are rare, e.g., ≤100 reads and ≤5% of the major form, than in the case in which both forms are known (*A*). However, the absolute number of pairs in which the minor form is not rare is almost twice the number of junctions annotated in WormBase (6782 versus 3428), and the other two cases (*C*,*D*) add another 4527 relatively well represented alternatively spliced junctions not in WormBase. The rare junctions could represent splicing errors, but considering their overlap in representation with junctions annotated in WormBase, they could also be biologically important.

Only a small fraction of the alternative splice junction pairs that we identified had both junctions annotated in WormBase (4107/5663 or 73% of pairs) and some were rare, raising the possibility that they resulted from splicing errors instead of biologically relevant events. To compare the pairs in which both the major and minor forms were annotated in WormBase (representing a curated set) to those with either one or both members of the pair newly detected in our data (novel) but overlapping a WormBase gene, we plotted the ratio of each minor form to its major form against its overall representation in our data sets, plotting separately the different classes of pairs (Fig. 2). As might be expected, the representation of the minor forms is comparatively higher if the junctions were previously known, e.g., 83.5% (3428/4107) of the minor form of examined pairs were represented by more than 100 reads in our data set or constituted >5% of the major form (Fig. 2A). In contrast, in pairs in which only the major form was previously known, only 14.6% (6782/46,484) met these criteria, although the absolute number of such junctions was larger (Fig. 2B). The pairs in which both junctions were novel (Fig. 2C) or the major form was novel (Fig. 2D) are more similar to junctions where both forms are known (72.9% [3714/5095] and 86.8% [813/937], respectively) and provide additional pairs in which the minor form is well represented and/or a substantial fraction of the major form. The relative abundance of these novel junctions compared to the curated WormBase annotations suggests they are biologically important events that are not represented in the curated set. The role of the rarer forms is less clear. However, given that for 16.5% of the curated pairs, the minor form is relatively rare (≤100 reads and <5% of the major form), we cannot rule out that the many additional rarer junctions are biologically significant. One explanation for these pairs in which the minor form is relatively rare is tissue-specific alternative splicing. As more data are collected from specific tissues, the functional importance of these forms should become clearer (Supplemental Fig. S12).

**Figure 3.**
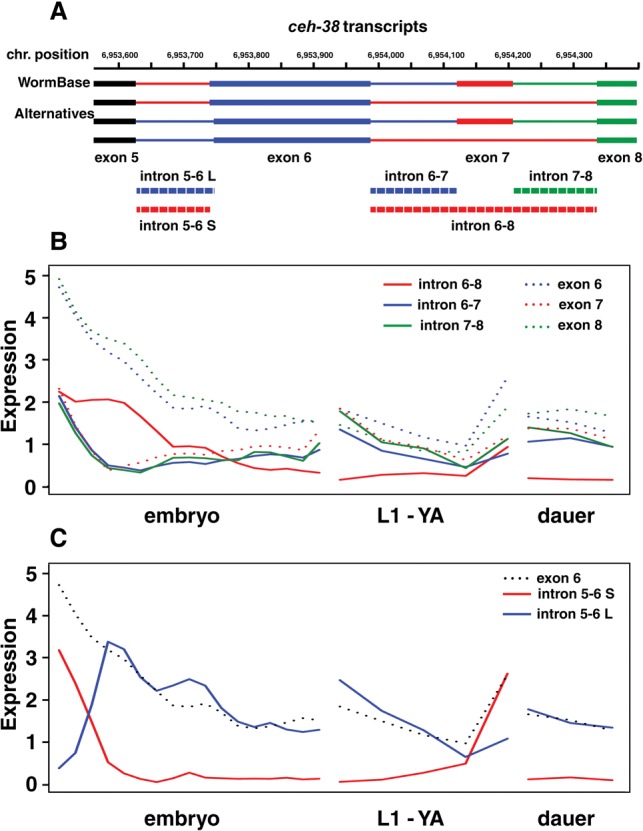
Alternative splicing in exons 5–9 of the transcription factor gene *ceh-38*. (*A*) In WormBase, only the topmost gene model is represented, using introns 5-6 S, 6-7, and 7-8 as illustrated. Our data show the presence of additional introns 5-6 L and 6-8. The former deletes two amino acids from exon 6, changing the spacing between the cut (in exon 5) and homebox (in exon 6) DNA binding domains. Intron 6-8 skips exon 7, but maintains the reading frame. (*B*) Expression data in dcpm for introns 6-7, 7-8, and 6-8 as well as exons 6, 7, and 8 indicate that although the included form is maternally inherited and then lost rapidly, the skipped form is expressed in the early zygote as well as maternally inherited. (*C*) Expression data in dcpm for introns 5-6 S and 5-6 L show that the shorter intron is maternally inherited and degraded rapidly. In contrast, the longer form has little maternal contribution, rises rapidly in the early embryo, and persists into the later embryo and larval stages, albeit at lower levels.

Our data thus reveal many more possible transcripts than are annotated in WormBase gene models. Further, the expression data for each intron across all the samples as well as exon expression data suggest in some cases particular stages at which these alternative forms may be important (Supplemental Tables S9, S10). For example, for the gene *ceh-38*, a broadly expressed transcription factor of the homeodomain class, WormBase shows exons 6, 7, and 8 as constitutively expressed with exon 5. Our data reveal that exon 6 has a second form with two additional amino acids at its start, altering the spacing between the cut and homeobox domains, and also that exon 7 can be skipped while maintaining the reading frame (Fig. 3A). The expression data for these different junctions and their flanking exons (Fig. 3B,C) show that although isoforms lacking or including exon 7 are both maternally expressed, the skipped form is present in early zygotes and is largely lacking in late embryogenesis and in the dauer stages. In contrast, the included isoform disappears rapidly in early embryogenesis and then slowly accumulates in later embryogenesis. Similarly, the two versions of exon 6 show distinct expression patterns, with one largely maternal and the other largely zygotic, and one present in the dauer and the other largely absent. Comparison of the patterns across the two different sets of alternative splices for *ceh-38* also suggests that the two alternative splices are used independently. Analyzing the expression of the minor and major alternative splice sites across the life cycle reveals hundreds of other examples of differential usage (Supplemental Table S8). Tissue-specific expression data will undoubtedly reveal many more.

### Operons

About 70% of *C. elegans* transcripts are *trans*-spliced, with the SL1 splice leader used for genes with independent promoters and the SL2 class of splice leaders used for downstream (internal) genes within operons (Blumenthal 2012). Notably, transcripts from some downstream genes in operons contain a mix of SL2 and SL1 splice leaders, suggesting that in these cases, the downstream gene is transcribed both as part of an operon and from its own promoter (Whittle et al. 2008). To test this supposition and to identify operons with internal promoters, we investigated the relationship between SL1 and SL2 usage and chromatin marks at downstream genes, using the splice leader data for each transcript.

**Figure 4.**
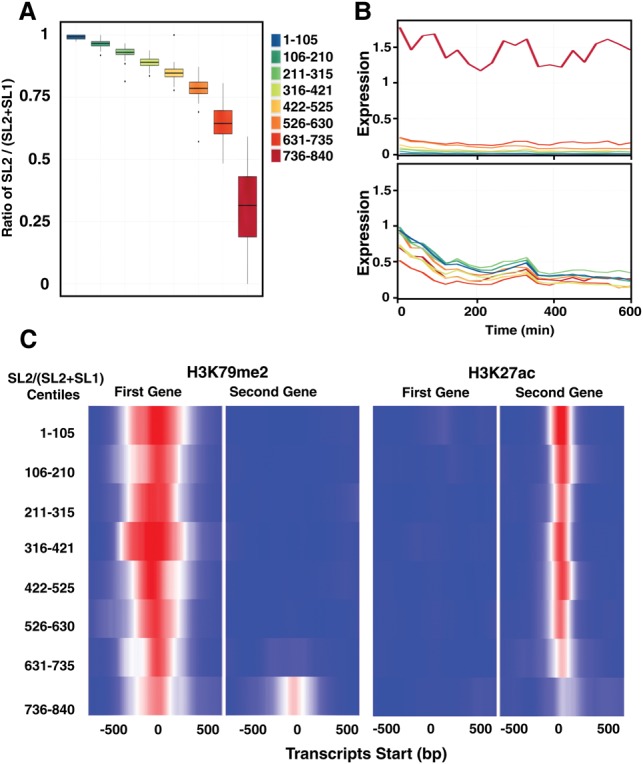
Operon gene regulation across development. (*A*) A box plot of the ratio of SL2 reads to all splice leader reads for all second genes in operons across the embryonic time series 0223. Well-expressed second genes in operons (dcpm ≥0.1) were divided into eight equally sized bins (105 genes) based on the overall SL2 fraction. Outliers may indicate stage-specific usage of the two promoters. (*B*) The same eight bins showing average SL1 (*top*) and SL2 (*bottom*) expression in dcpm across development for the second genes. (*C*) Average H3K27ac signal across the transcript start site of the first (*left*) and second (*right*) gene in operons, divided into the same eight bins as in *A*. Marks of open chromatin may serve to maintain open chromatin for polymerase read-through during transcription of the operon. Average H3K79me2 signal across the transcription start site of the first (*left*) and second (*right*) gene in operons, divided into the same eight bins as in *A*. Promoter areas of these second genes are sites of active regulation.

The second genes in operons vary widely in the proportion of SL2 usage (Fig. 4A). Most downstream genes have predominantly SL2 splice leaders, but a subset of about 100 operons have SL1 as the majority leader. This increased ratio is not at the expense of SL2 usage, but rather reflects an increase in SL1 usage, consistent with the supposition of expression from an independent promoter (Fig. 4B). We also found a slight increase in the average distance between the first and second genes for the quantile with high SL1 usage for the second gene and slightly less correlation in the expression level between the first and second gene (Supplemental Fig. S13).

Because different chromatin marks are associated with specific functional features of genes, we next examined chromatin profiles in published ChIP-seq data with the transcript start site of the first and second genes in operons (Ercan et al. 2011; Liu et al. 2011). Those second genes that had SL1 as the major splice leader had patterns similar to the patterns of the first genes. For example, the histone mark H3K79me2 is bound across the start of the 100 genes with the highest SL1 ratios (Fig. 4C) with similar findings for other promoter associated marks (H3K36me1/2/3, H3K79me1/2/3, H3K4me2/3, H4K8ac, H4K16ac, H4tetraac, HTZ-1) and heterochromatin (H3K9me1/2/3), indicating the promoter areas of these second genes are sites of active regulation (Supplemental Fig. S14). Interestingly, for second genes with SL2 as the major form, the H3K27ac signal was very strong over the transcript start site (as was the RNA Pol II signal), whereas the signal was lacking entirely over start sites of the first genes and the second genes with SL1 as the major splice form. These marks of open chromatin perhaps serve to maintain open chromatin for polymerase read-through during transcription of the operon.

### Histones

Histone gene expression is likely to be a substantial component of overall gene expression in a rapidly dividing embryo like *C. elegans*. However, because replicative histones, i.e., those histones incorporated during DNA replication, lack poly(A) tails, their mRNAs are severely underrepresented in poly(A)-selected or oligo-dT-primed cDNA libraries. Also, each of the core histones is present in 14 or 15 almost identical copies, complicating the interpretation of microarray data. Because we used a ribosomal rRNA depletion method and random priming in three of our synchronized embryonic series and in selected post-embryonic stages, the histone mRNAs are faithfully represented in those samples. To quantify histone gene expression as fully as possible, we identified bases at which the individual copies differed from one another to aid in assigning reads, and failing that, we distributed reads equally between identical copies to calculate expression levels.

**Figure 5.**
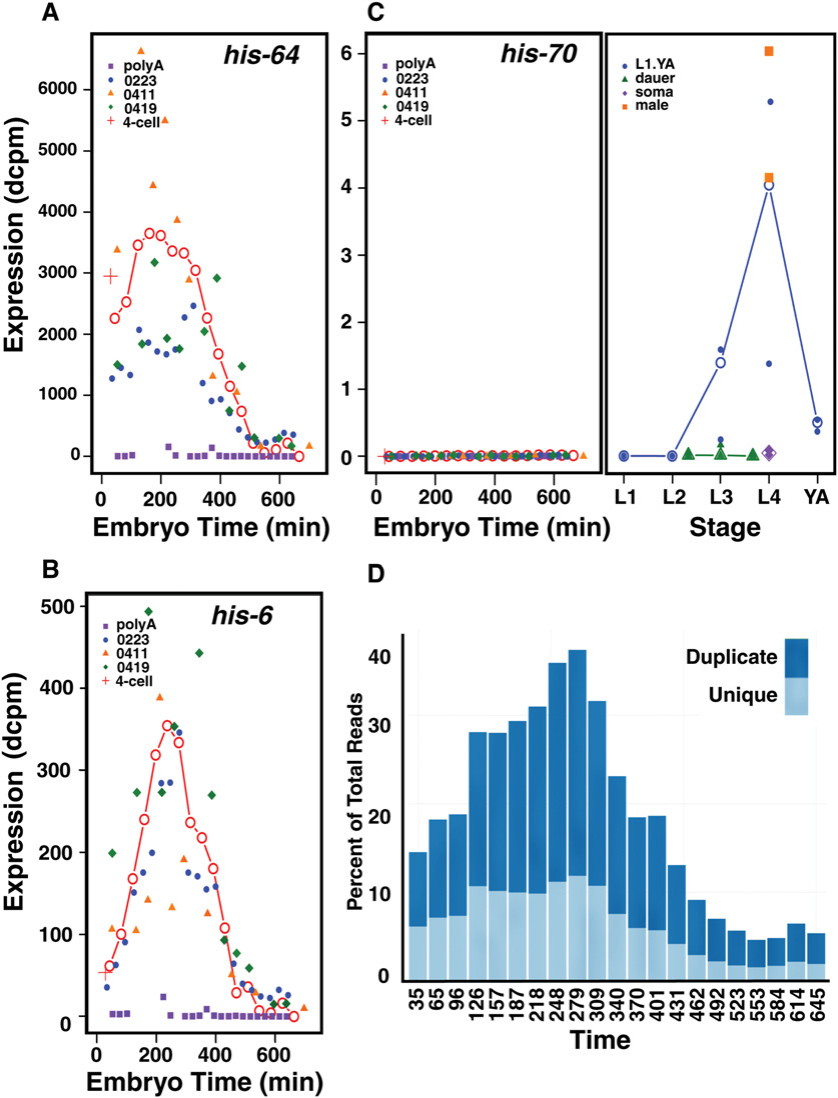
Histone expression across development. (*A*) The expression in dcpm of the replicative histone, *his-64*, an example of a pattern with a substantial maternal component. The expression is shown across embryogenesis (labeled in embryo time) for the unified series (red line, open circles), the three rRNA subtracted series (blue, orange, and green), and the poly(A)-selected series (purple) along with the single four-cell sample (red cross). Its pattern is typical of the genes located in the clusters on Chromosome IV. (*B*) The expression in dcpm of the replicative histone, *his-6*, an example of a largely zygotically expressed histone. The pattern is shown across the same time points, colored as in *A*. Its expression pattern is typical of the histone clusters on Chromosomes I and V. (*C*) The expression in dcpm of *his-70*, a C-terminally truncated H3.3 variant (Ooi et al. 2006), is shown across embryogenesis (*left*) and into larval and adult time points (*right*). The gene lacks an intron but apparently has a poly(A) tail. The embryogenesis time points are colored as in *A*, the late time points are for L1 through young adult (blue), the dauer stages (green), soma (purple), and male (orange). Both the individual samples (closed symbols) and weighted averages (open symbols) are shown. Post-embryonic time points are shown in approximate chronological order. (*D*) The percentage of total aligned sequence reads specific to histone genes during embryonic development (time in minutes) for the 0223 rRNA subtracted series (0223) is shown. Uniquely aligned reads are shown in light blue, whereas sequence reads aligning equally to multiple histone gene family members are shown in dark blue.

Inspection of the resultant data shows two major expression patterns for the replicative histone genes with patterns consistent for all the genes in a cluster. The *C. elegans* core replicative histones are aggregated into seven clusters (Supplemental Table S11), with each cluster containing one or more sets of each of the four core histones (H2B:H2A—H3:H4, where “:” denotes head to head orientation). The two clusters on Chromosome IV show substantial levels at the earliest embryonic time points, which then rise further to a peak at about 200–250 min (Fig. 5A). There are substantial levels in the dissected gonad sample as well (also prepared with the rRNA depletion method), consistent with a maternal origin for these messages. Because excess histone protein is generally detrimental to cells (Gunjan and Verreault 2003; Kurat et al. 2014), these messages may be subject to translational control. The mRNAs from other clusters on Chromosomes I and V have largely zygotic expression patterns, present at relatively low levels in the first embryonic samples, rising rapidly to a peak at ∼200–300 min, and then falling rapidly (Fig. 5B).

We looked for sequence motifs in the short intergenic regions between head-to-head oriented genes that might correlate with two different expression patterns. We readily found two previously described motifs (Roberts et al. 1987, 1989) and one other shorter motif that were also present in the homologous *C. briggsae* regions, but failed to find any specifically associated with either the zygotic or maternal patterns (Supplemental Fig. S15).

The replacement H3.3 histone and its variants, i.e., those histones incorporated outside of DNA replication, are expressed in a variety of patterns, with *his-74* having an early maternal pattern, *his-72* an early zygotic, and *his-71* a late zygotic pattern. Strikingly, *his-70*, encoding a C-terminally truncated H3.3 protein (Ooi et al. 2006), is expressed almost exclusively in males and in L4 animals, which also make sperm (Fig. 5C). Perhaps, *his-70* functions similarly to sperm-specific histones in other organisms.

In addition to their patterns, the histones in the total RNA samples are notable for their high levels of expression, with 22 of the top 25 expression values across all stages derived from histone genes (the other three are ribosomal proteins). Intrigued by this finding, we looked at the representation of histone sequence reads as a fraction of total aligned reads at each stage. Remarkably, at the peak around 300–325 min, >35% of all aligned reads derive from histone genes (Fig. 5D). Because histone mRNAs are relatively short, this level implies that an even larger fraction of mRNA molecules derive from histone genes. The maximal levels occur as the embryo is entering into the last major round of cell division, creating a demand for 8 million histone proteins in less than an hour for each of about 250 dividing cells.

### Noncoding RNAs and pervasive transcription

Our RNA-seq data also assay the expression of other transcripts, including annotated WormBase noncoding RNAs (>100 bp) and novel transcripts detected through splice junctions outside annotated WormBase transcripts. Other aligned reads are scattered across the genome, possibly representing rare, novel transcripts; but these reads could also derive from low levels of DNA contamination.

Some of the WormBase ncRNAs are expressed at levels comparable to moderately expressed protein coding genes, and many of these are differentially expressed (Supplemental Tables S12, S13). Taking the 170 lincRNAs as a well-defined example set (Nam and Bartel 2012), starting from reads spanning a splice junction, we were able to build gene models around 139 of these that overlapped the WormBase model. However, based on these transcript models, we found that 73 of the lincRNAs have open reading frames (ORFs) of greater than 80 amino acids. Some 32 of these have significant matches against the NCBI nonredundant protein database. In addition, 14 others could be linked to WormBase protein coding genes via novel splice junctions and exons, suggesting they represent previously undetected UTRs.

The splice junctions that fall outside of WormBase-annotated genes could derive from additional previously undetected ncRNAs. As described above, of the 17,472 splice junctions that failed to overlap WormBase genes, 6642 junctions could not be linked to previously annotated transcripts. Of these, all but 859 were flanked by RNA-seq reads and could be used to build new gene models. Some 3659 of these junctions fell in models that had ORFs of more than 80 amino acids and may represent previously undetected protein coding genes. The remaining junctions in models with ORFs of up to 80 amino acids (2124) are only poorly represented in our data sets, with only ∼4% of them having more than 30 reads spanning the junction (0.005 per million reads). Nonetheless, these junctions, especially the more highly expressed junctions, may identify additional ncRNAs.

We looked more broadly at transcription outside the annotated regions and outside our novel gene models. We began by looking for contiguous blocks of read coverage as a function of size and levels of read coverage (Supplemental Table S14). For example, in the aggregate data set, we find 1034 blocks of greater than 200 bases with an expression level of 0.01 dcpm or greater (equivalent to an FDR of 0.05), and fewer than half of these are greater than 300 bases in length. Almost all of these are poorly expressed as shown by the rapid fall off in numbers with increasing thresholds. Because expression in the aggregate data set may mask stage-specific transcripts, we also did a similar analysis across the individual samples. Looking across multiple samples increases the chance of false discoveries but does provide an upper bound on the estimate of additional coding regions. Looking across the embryonic samples, we identified 4805 blocks of above-threshold coverage of greater than 200 bases that were shared by at least two samples and 3025 blocks shared by at least five samples. Again, almost all of these blocks are poorly expressed. More directed experimental work will be required to determine the role of these transcribed regions.

We also asked whether the signals in these intergenic regions correlated with expression of either coding or noncoding RNAs. Using a window size of 80 kb and looking across the genome, we find several regions that appear to have higher or lower expression. These regions correlate significantly with levels of protein coding gene expression for each of the chromosomes but only weakly with ncRNA gene expression (Table 1; Supplemental Fig. S16). The correlation with protein coding gene expression does not appear to be attributable to undetected 5′ or 3′ UTRs, since looking across the protein coding genes in aggregate we saw no evidence for extended transcription (Supplemental Fig. S17). The correlation with protein coding gene expression suggests that regions with higher levels of pervasive transcription could reflect simply greater access of the polymerase to the DNA; alternatively, some of the transcription could result from enhancer sequences associated with the protein coding genes.

**Table 1.**
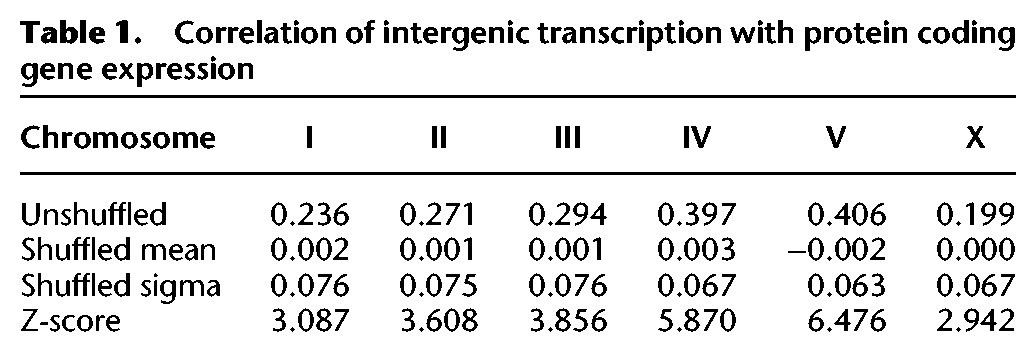
Correlation of intergenic transcription with protein coding gene expression

## Discussion

As part of the modENCODE Project, we generated deep RNA-seq data sets for *C. elegans* using a consistent methodology across the life cycle, including a detailed embryonic time course. These data sets provide the community with information about the patterns of protein coding gene and ncRNA expression as well as splice junction and splice leader usage. Our analysis of these data uncovered patterns and features of interest, but greater value will accrue as the community utilizes the expression patterns to accelerate research on *C. elegans*.

The expression patterns of the protein coding genes provide new insights and confirm and extend earlier results. Males show a large number of genes with maximal expression, and a very large fraction of these are not detected in any hermaphrodite stage or are present predominantly at the L4 stage. These latter likely represent sperm-specific proteins but could also be L4-specific proteins. The genes detected only in males likely represent genes used only in the generation of male specific traits. About half the genes with their maximal expression in the earliest embryo time point show high expression in the young adult samples, documenting their maternal origins. The majority of these are lost rapidly in the subsequent embryo samples, but others show a much slower decay. Similarly, the genes that show maximal expression in the young adult stage also frequently are present in the first embryo stage; their relatively lower embryonic levels suggest these are mRNAs involved in the making of the oocyte itself that persist into the embryo. The three dauer stages (entry, dauer, and exit) are also striking, with a large number of genes maximally expressed in one of the dauer stages. These genes tend to be strongly expressed as well in the other dauer stages.

Looking at gene expression dynamics during embryogenesis shows that although many genes are falling in expression early and many others are rising either early or late, there is a paucity of genes changing in either direction from ∼200 to 400 min. This period largely overlaps the period of maximal histone gene expression, in which at the peak almost 40% of mapped sequence reads derive from histone mRNAs. Presuming that histone mRNAs are translated with an efficiency similar to poly(A)^+^ mRNAs, almost half the translational capacity of the embryo is devoted to DNA replication and chromatin formation needed to achieve the rapid division of its increasing number of cells. It is tempting to speculate that the high demands for cell division limits the ability of the embryo to initiate new gene expression patterns, but the results are also consistent with a program of early fate commitment followed by a proliferation of these precursors before terminal differentiation ensues. The very large fraction of translational capacity devoted to cell division also suggests that the worm embryo may be at the practical limits of cell division speed and that these limits place strong constraints on the genome size. A 10% increase in genome size would in turn demand a 10% increase in histone production in a very short time window.

We find many more splice junctions than are currently incorporated into WormBase gene models. Most of these extra junctions either overlap WormBase models or can be linked to them via other new junctions and plausible exons. The bulk of these overlapping junctions share either a donor or acceptor site (or both) with WormBase junctions and thus represent potential alternative splices forms. Some of these new, alternative junctions are expressed at levels comparable to the WormBase annotated junctions, but others are represented at much lower levels. These low relative levels could be the result of weak or cryptic splice signals recognized by the splicing machinery. However, several observations suggest that many of these poorly expressed junctions could be biologically important alternative forms. In an organism with 959 somatic cells and many cell types represented with only one or a few cells, cell-specific isoforms would be expected to be present at only 1% or less of the major form. As RNA-seq data becomes available for an increasing number of different tissues and cell types, the number of alternative junctions with differential expression will surely increase. These data should provide a rich source for the community for future discovery.

Our data sets also shed light on the expression of noncoding RNAs and what has been characterized as “pervasive” or “background” transcription. Our splice junction data links several annotated ncRNAs to protein coding genes, and a significant fraction of the remainder contain open reading frames up to 80 amino acids. The ncRNAs with which we can associate protein coding functions account for the bulk of the well-expressed annotated ncRNAs. Similarly, of the 6642 splice junctions that fall outside of protein coding genes and are not linked to them by other splice junctions, 3659 are associated with gene models that have open reading frames of up to 80 amino acids. Nonetheless, the remaining 2124 junctions provide the community with possible ncRNAs whose expression patterns are now known, but whose functions are unclear.

Outside the annotated genes and the models built around splice junctions, we find additional blocks of transcribed sequence. Although relatively small in number, these above-threshold regions also remain unexplained. By looking at larger windows (80 kb) across the genome, we do find these blocks correlated with protein coding gene expression. Perhaps the open chromatin associated with blocks of well-expressed protein coding genes predisposes other DNA in the region to assemble the transcription machinery. Alternatively, larger repressed regions may exclude the transcriptional machinery. The level of nonspecific transcription in these regions is very low, and DNA contamination remains a possible artifactual cause of the signal. But if DNA contamination is the source, the finding of regional specificities would require some other explanation of the correlation with protein coding gene expression levels. The cost of this “background” transcription is low compared to the energy spent in transcribing introns, and this low level may represent the limits of selective pressure to evolve a more precise system.

The associations we find between the ratios of the two spliced leader sequences and different chromatin marks provide further evidence that the ratios can reliably be used as a proxy for the existence of an independent promoter at internal genes of operons (Ooi et al. 2006; Allen et al. 2011). The inverse correlation of the H3K27ac mark with independent promoters is unexpected, because that mark is often associated with promoters of highly expressed protein coding genes. What signal localizes the mark in operons without an independent promoter is unclear.

Our data sets, covering the full life cycle of the hermaphrodite, including the dauer stages as well as young males, provide a rich catalog for the community. They provide a comprehensive picture of the transcripts present in the whole animal at each stage of the life cycle. The expression data can be used both to support and rule out possible regulatory and other genetic interactions. However, our data do not provide information about the spatial constraints on expression. An obvious next step is to obtain RNA-seq from specific tissues and cells. The ultimate goal would be the RNA content of every cell throughout development. The goal for *C. elegans* should be nothing less than a complete knowledge of the RNAs present in each cell through development. Such a catalog would form the foundation for a comprehensive understanding of the regulatory networks that dictate the emergence of the moving worm obtained solely from the information contained in the genome.

## Methods

### Embryo growth and isolation

Large populations of synchronized embryos were generated by successive rounds of bleaching. In the first round, eggs were collected from gravid adults and hatched in the absence of food to produce synchronized L1s. In the second round, eggs were collected from young adults as soon as eggs were detected in some worms. Again the eggs were hatched in the absence of food and produced a more highly synchronized population of L1s. In the third round, eggs were again collected from young adults as soon as eggs were detected in some worms. This extra round yielded a higher fraction of young adults with eggs, and the eggs showed tighter synchrony.

### Post-embryonic staging

The samples for post-embryonic samples were as previously reported (Hillier et al. 2009; Gerstein et al. 2010, 2014). Exact times for growth after plating of starved L1s (all at 25°C) were as follows: (1) L1: worms were grown 4.0 h post-L1 plating; (2) L2: for 17.75 h (Pn.p cells visible but not divided, gonad just starting to proliferate); (3) L3: for 26.75 h (Pn.p cells divided once or twice; gonad just starting to turn up); (4) L4: for 34.25 h (vulvae are in Christmas tree stage, gonad has passed bend, sperm are present); (5) young adult: for 46 h (vulvae fully formed and oocytes present in gonad, but no embryos); (6) dauer entry: *daf-2(e1370)* 48 h post-L1 stage larvae; (7) dauer: *daf-2(e1370)* 91 h post-L1 stage larvae; and (8) dauer exit: *daf-2(e1370)* at 25°C for 91 h and at 15°C for 12 h; male L4: *him-8(e1480)* mid-L4 30 h post-L1 stage larvae (filtered through mesh to purify males); soma: JK1107(*glp-1(q224)*) mid-L4 30 h post-L1 stage larvae. Dissected gonads were from N2 (wild type) animals grown for 48 h at 20°C post-L1 stage larvae; approximately 200 gonads dissected and isolated from carcasses.

### RNA isolation and library construction

Total RNA was prepared as previously described with minor modifications (Hillier et al. 2009). Ribosomal subtraction was performed using Ribozero kits (Epicentre) according to the manufacturer's instructions. cDNA was generated, and RNA-seq libraries were prepared as previously described with minor modifications (Hillier et al. 2009). See Supplemental Methods for details.

### Unification of embryonic time series samples

A single unified expression time series based on a standard developmental time scale was created from the multiple replicate experimental time series using a Bayesian statistical model (Supplemental Methods). Using the 6000 most highly expressed genes, four parameters are inferred for each of the four experimental time series: (1) initial mean standard developmental time of the population of embryos; (2) growth rate of the population of embryos, compared to the standard time scale; (3) initial distribution of developmental stages in the population; and (4) increase in the variance of the initial stage distribution over the time of the experiment. From these inferred parameters, the stage composition of each of the experimental samples was calculated. The different time series were then unified by deconvolving to a single time series using a similar Bayesian model and the Metropolis-Hastings algorithm. The parameters inferred for this second phase model are the gene expression values for each of the 20,000 genes in WormBase in each of the individual developmental stages. To convert the pseudotime values to standard developmental times, the nuclear counts for individual eggs for the 0 min sample in the 0223 series were converted into developmental times (after the division into two cells) and the developmental times averaged to estimate the sample time.

### Alignment/expression quantification

Reads were aligned against the *C. elegans* genome (WS220) using cross_match (P Green, unpubl.) and against a set of *C. elegans* transcript models (Gerstein et al. 2010). Methods for defining whether SLs, poly(A)s, and splice junctions met false positive/false discovery rate thresholds are described (Hillier et al. 2009). Each transcribed unit was assigned an expression level by its average depth of coverage per base per million reads (dcpm) (Hillier et al. 2009).

### Differential expression analysis using edgeR

Read counts per gene were used as input to edgeR to identify those genes that were up- and down-regulated between developmental stages. In each case, biological replicate pairs (as defined by the Spearman correlation of pairs of samples) (Supplemental Methods) were used. For each comparison, we only included the genes that had a dcpm of at least 0.07 in at least one of the samples used to increase the statistical power of the analysis of differential expression (Anders et al. 2013).

### GO analysis

Each set of up- and down-regulated genes were examined for enrichment of gene ontology (GO) terms using the online GO database GOminer (Zeeberg et al. 2003). Terms were clustered using Ward minimum variance hierarchical clustering based on enrichment for each gene set.

### Change point analysis

A Bayesian statistical model was used to detect change points in the unified embryonic gene expression time series. A reversible jump Monte Carlo Markov Chain (MCMC) algorithm (Green 1995) infers the number and location of the change points in developmental time. For each gene, the number of change points was limited to less than three. The generative model assumes that the time series expression is a linear function of time and the slope of that function changes at the change points. This model results in a piecewise linear function, representing the unified expression time series for each gene measured, with one to four possible segments. To prevent overfitting, an exponential distribution was used as the prior on the number of change points.

### Differential intron usage

To find introns used differentially during the course of the life cycle, we first identified all sites with two or more junctions arising from them (alternatively spliced) where the minor form was represented by at least 10 spanning reads and was present at 1% or more of the major form. To find those introns that were differentially used during the lifecycle, we calculated the normalized ratio of the minor form to the total of the minor plus major form and looked for consecutive samples, allowing for up to two exceptions, in which the ratio was either unusually high (>0.85; minor form predominating) or low (<0.15; major form predominating). For each site, we report the top two runs above 0.85 (Supplemental Table S8a) and the top two runs below 0.15 (Supplemental Table S8b). We also generated graphs for each pair, showing the relative expression of each junction for each of the samples as well as the normalized ratio of the minor form to the total (available at http://genome.sfu.ca/gexplore/gexplore_search_expression.html).

### Operons

Operon annotation was obtained from WormBase build WS220. The ratio of SL2 over total SL dcpm [SL2/(SL1 + SL2)] was calculated for each gene at each time point in every operon for the samples of the 0223 series. Operons were then ranked based on this ratio for the second gene in the operon averaged across the first seven time points or 3 h of development (Supplemental Methods). Operons were then placed in eight bins consisting of 105 genes each for further analysis. We also mined existing chromatin data sets, calculating the average signal in the 1000 bases surrounding the TSS of both first and second genes.

### Pervasive transcription

Each chromosome was divided into non-overlapping 80-kb regions. Each base was annotated as intergenic, coding, or noncoding, and a single vector of dcpm values was calculated for each chromosome and each annotation type (Supplemental Methods). The vectors were compared by calculating the Spearman correlation for each pair of annotated features. To assess the value of the random correlation between the pairs of annotated features, the intergenic vector is randomly permuted 1000 times, and the permuted intergenic vector is correlated with the coding vector. The mean and the standard deviation of the 1000 Spearman correlation values is calculated and used to calculate the standard *Z*-score of the Spearman correlation values.

## Data access

The sequencing data from this study have been submitted to the NCBI Sequence Read Archive (SRA; http://www.ncbi.nlm.nih.gov/sra/) under the accession numbers listed in Supplemental Table S1.

## Supplementary Material

Supplemental Material

## References

[BOECKGR202663C29] Allen MA, Hillier LW, Waterston RH, Blumenthal T. 2011 A global analysis of *C. elegans trans*-splicing. Genome Res 21: 255–264. 21177958 10.1101/gr.113811.110 PMC3032929

[BOECKGR202663C1] Anders S, McCarthy DJ, Chen Y, Okoniewski M, Smyth GK, Huber W, Robinson MD. 2013 Count-based differential expression analysis of RNA sequencing data using R and Bioconductor. Nat Protoc 8: 1765–1786. 23975260 10.1038/nprot.2013.099

[BOECKGR202663C2] Baugh LR, Hill AA, Slonim DK, Brown EL, Hunter CP. 2003 Composition and dynamics of the *Caenorhabditis elegans* early embryonic transcriptome. Development 130: 889–900. 12538516 10.1242/dev.00302

[BOECKGR202663C3] Blumenthal T. 2012 Trans-splicing and operons in *C. elegans*. In WormBook (ed. The *C. elegans* Research Community, WormBook), pp. 1–11. 10.1895/wormbook.1.5.2 http://www.wormbook.org/.

[BOECKGR202663C4] Broitman-Maduro G, Owraghi M, Hung WW, Kuntz S, Sternberg PW, Maduro MF. 2009 The NK-2 class homeodomain factor CEH-51 and the T-box factor TBX-35 have overlapping function in *C. elegans* mesoderm development. Development 136: 2735–2746. 19605496 10.1242/dev.038307 PMC2730403

[BOECKGR202663C5] Ercan S, Lubling Y, Segal E, Lieb JD. 2011 High nucleosome occupancy is encoded at X-linked gene promoters in *C. elegans*. Genome Res 21: 237–244. 21177966 10.1101/gr.115931.110 PMC3032927

[BOECKGR202663C6] Francesconi M, Lehner B. 2014 The effects of genetic variation on gene expression dynamics during development. Nature 505: 208–211. 24270809 10.1038/nature12772

[BOECKGR202663C7] Gerstein MB, Lu ZJ, Van Nostrand EL, Cheng C, Arshinoff BI, Liu T, Yip KY, Robilotto R, Rechtsteiner A, Ikegami K, 2010 Integrative analysis of the *Caenorhabditis elegans* genome by the modENCODE project. Science 330: 1775–1787. 21177976 10.1126/science.1196914 PMC3142569

[BOECKGR202663C8] Gerstein MB, Rozowsky J, Yan KK, Wang D, Cheng C, Brown JB, Davis CA, Hillier L, Sisu C, Li JJ, 2014 Comparative analysis of the transcriptome across distant species. Nature 512: 445–448. 25164755 10.1038/nature13424 PMC4155737

[BOECKGR202663C9] Green PJ. 1995 Reversible jump Markov chain Monte Carlo computation and Bayesian model determination. Biometrika 82: 711–732.

[BOECKGR202663C10] Gunjan A, Verreault A. 2003 A Rad53 kinase-dependent surveillance mechanism that regulates histone protein levels in *S. cerevisiae*. Cell 115: 537–549. 14651846 10.1016/s0092-8674(03)00896-1

[BOECKGR202663C11] Hashimshony T, Feder M, Levin M, Hall BK, Yanai I. 2015 Spatiotemporal transcriptomics reveals the evolutionary history of the endoderm germ layer. Nature 519: 219–222. 25487147 10.1038/nature13996 PMC4359913

[BOECKGR202663C12] Hillier LW, Reinke V, Green P, Hirst M, Marra MA, Waterston RH. 2009 Massively parallel sequencing of the polyadenylated transcriptome of *C. elegans*. Genome Res 19: 657–666. 19181841 10.1101/gr.088112.108 PMC2665784

[BOECKGR202663C13] Kim SK, Lund J, Kiraly M, Duke K, Jiang M, Stuart JM, Eizinger A, Wylie BN, Davidson GS. 2001 A gene expression map for *Caenorhabditis elegans*. Science 293: 2087–2092. 11557892 10.1126/science.1061603

[BOECKGR202663C14] Krause M, Liu J. 2012 Somatic muscle specification during embryonic and post-embryonic development in the nematode *C. elegans*. Wiley Interdiscip Rev Dev Biol 1: 203–214. 23801436 10.1002/wdev.15 PMC3880184

[BOECKGR202663C15] Kurat CF, Recht J, Radovani E, Durbic T, Andrews B, Fillingham J. 2014 Regulation of histone gene transcription in yeast. Cell Mol Life Sci 71: 599–613. 23974242 10.1007/s00018-013-1443-9 PMC11113579

[BOECKGR202663C16] Levin M, Hashimshony T, Wagner F, Yanai I. 2012 Developmental milestones punctuate gene expression in the *Caenorhabditis* embryo. Dev Cell 22: 1101–1108. 22560298 10.1016/j.devcel.2012.04.004

[BOECKGR202663C17] Liu T, Rechtsteiner A, Egelhofer TA, Vielle A, Latorre I, Cheung MS, Ercan S, Ikegami K, Jensen M, Kolasinska-Zwierz P, 2011 Broad chromosomal domains of histone modification patterns in *C. elegans*. Genome Res 21: 227–236. 21177964 10.1101/gr.115519.110 PMC3032926

[BOECKGR202663C18] McGhee JD, Fukushige T, Krause MW, Minnema SE, Goszczynski B, Gaudet J, Kohara Y, Bossinger O, Zhao Y, Khattra J, 2009 ELT-2 is the predominant transcription factor controlling differentiation and function of the *C. elegans* intestine, from embryo to adult. Dev Biol 327: 551–565. 19111532 10.1016/j.ydbio.2008.11.034 PMC2706090

[BOECKGR202663C19] Nam JW, Bartel DP. 2012 Long noncoding RNAs in *C. elegans*. Genome Res 22: 2529–2540. 22707570 10.1101/gr.140475.112 PMC3514682

[BOECKGR202663C20] Ooi SL, Priess JR, Henikoff S. 2006 Histone H3.3 variant dynamics in the germline of *Caenorhabditis elegans*. PLoS Genet 2: e97. 16846252 10.1371/journal.pgen.0020097 PMC1484599

[BOECKGR202663C21] Raj A, Rifkin SA, Andersen E, van Oudenaarden A. 2010 Variability in gene expression underlies incomplete penetrance. Nature 463: 913–918. 20164922 10.1038/nature08781 PMC2836165

[BOECKGR202663C22] Roberts SB, Sanicola M, Emmons SW, Childs G. 1987 Molecular characterization of the histone gene family of *Caenorhabditis elegans*. J Mol Biol 196: 27–38. 3656446 10.1016/0022-2836(87)90508-0

[BOECKGR202663C23] Roberts SB, Emmons SW, Childs G. 1989 Nucleotide sequences of *Caenorhabditis elegans* core histone genes: Genes for different histone classes share common flanking sequence elements. J Mol Biol 206: 567–577. 2544730 10.1016/0022-2836(89)90566-4

[BOECKGR202663C24] Robinson MD, McCarthy DJ, Smyth GK. 2010 edgeR: a Bioconductor package for differential expression analysis of digital gene expression data. Bioinformatics 26: 139–140. 19910308 10.1093/bioinformatics/btp616 PMC2796818

[BOECKGR202663C25] Shin H, Hirst M, Bainbridge MN, Magrini V, Mardis E, Moerman DG, Marra MA, Baillie DL, Jones SJ. 2008 Transcriptome analysis for *Caenorhabditis elegans* based on novel expressed sequence tags. BMC Biol 6: 30. 18611272 10.1186/1741-7007-6-30 PMC2474577

[BOECKGR202663C26] Whittle CM, McClinic KN, Ercan S, Zhang X, Green RD, Kelly WG, Lieb JD. 2008 The genomic distribution and function of histone variant HTZ-1 during *C. elegans* embryogenesis. PLoS Genet 4: e1000187. 18787694 10.1371/journal.pgen.1000187 PMC2522285

[BOECKGR202663C27] Yuzyuk T, Fakhouri TH, Kiefer J, Mango SE. 2009 The polycomb complex protein *mes-2/E(z)* promotes the transition from developmental plasticity to differentiation in *C. elegans* embryos. Dev Cell 16: 699–710. 19460346 10.1016/j.devcel.2009.03.008 PMC2693235

[BOECKGR202663C28] Zeeberg BR, Feng W, Wang G, Wang MD, Fojo AT, Sunshine M, Narasimhan S, Kane DW, Reinhold WC, Lababidi S, et al. 2003 GoMiner: a resource for biological interpretation of genomic and proteomic data. Genome Biol 4: R28. 12702209 10.1186/gb-2003-4-4-r28 PMC154579

