# Supplementary material for: The time-resolved transcriptome of C. elegans
Source: Genome Res. 2016 Oct;26(10):1441–50. doi: 10.1101/gr.202663.115 (PMC5052054; doi:10.1101/gr.202663.115)
Supplement: Supplemental Material [file supp_26_10_1441__index.html]

The time-resolved transcriptome of C. elegans — Supplemental Material 

# The time-resolved transcriptome of *C. elegans*

## Supplemental Material

- Supplemental\_Guide.txt
- Supplemental\_Methods.docx
- Supplemental\_Table\_S1.xlsx
- Supplemental\_Table\_S2.gz
- Supplemental\_Table\_S3.gz
- Supplemental\_Table\_S4.xlsx
- Supplemental\_Table\_S5.xlsx
- Supplemental\_Table\_S6.xlsx
- Supplemental\_Table\_S7.docx
- Supplemental\_Table\_S8a.txt.gz
- Supplemental\_Table\_S8b.gz
- Supplemental\_Table\_S9.gz
- Supplemental\_Table\_S10.gz
- Supplemental\_Table\_S11.xlsx
- Supplemental\_Table\_S12.gz
- Supplemental\_Table\_S13.gz
- Supplemental\_Table\_S14.xlsx
- Supplemental\_Fig\_S1.docx
- Supplemental\_Fig\_S2.docx
- Supplemental\_Fig\_S3.docx
- Supplemental\_Fig\_S4.docx
- Supplemental\_Fig\_S5.docx
- Supplemental\_Fig\_S6.docx
- Supplemental\_Fig\_S7.docx
- Supplemental\_Fig\_S8.docx
- Supplemental\_Fig\_S9.docx
- Supplemental\_Fig\_S10.docx
- Supplemental\_Fig\_S11.docx
- Supplemental\_Fig\_S12.docx
- Supplemental\_Fig\_S13.docx
- Supplemental\_Fig\_S14.docx
- Supplemental\_Fig\_S15.docx
- Supplemental\_Fig\_S16.docx
- Supplemental\_Fig\_S17.docx
