# Supplementary material for: The time-resolved transcriptome of C. elegans
Source: Genome Res. 2016 Oct;26(10):1441–50. doi: 10.1101/gr.202663.115 (PMC5052054; doi:10.1101/gr.202663.115)
Supplement: Supplemental Material [file supp_gr.202663.115_Supplemental_Methods.docx]

**Supplemental Methods**

**Embryo Growth and isolation**

Large populations of synchronized embryos were generated by successive rounds of bleaching. In the first step a mixed gravid adult population was treated with bleach solution to release embryos. The embryos were hatched overnight in the absence of food (S-basal) at 20° C with gentle shaking. Approximately 50,000 hatched L1 stage larvae were plated on 150 mm enriched peptone plates seeded with NA22 bacteria. The worms were grown at 20° C for about 48 hours to reach the adult stage. The young adult worms were collected and bleached to release embryos. The embryos were washed thoroughly to remove bleach solution, and were allowed to hatch overnight at 20° C with shaking in S-basal buffer. The newly hatched L1 worms were plated on enriched peptone plates seeded with NA22 bacteria and grown at 20° C for about 40 hours. Thereafter, the young adult worms were closely monitored for the presence of embryos. As soon as embryos were observed in a few worms, the adults were collected and bleached to collect early embryos. The synchronized early embryos were incubated in S-basal at 20° C with gentle shaking. The 0 time point sample was collected immediately and subsequent samples were collected every 30 minutes. For each time point approximately 80,000-100,000 embryos were collected and centrifuged to remove excess liquid. A small aliquot of embryos was fixed in Carnoy’s solution and stained with propidium iodide. Fixed, stained embryos were later photographed with a Zeiss confocal microscope, imported into Ace-Tree (Boyle et al. 2006) and the nuclei counted. The remaining embryos were resuspended in 300 ul of Trizol (Gibco), flash frozen in liquid nitrogen and stored at -80° C until the RNA isolation step.

**RNA isolation and library construction**

Total RNA isolation from the Trizol (Gibco) suspension was performed according to the manufacturer’s instructions. After three rounds of freeze/thawing and vortexing, the sample was extracted with 67 µl of chloroform. The collected aqueous phase was precipitated with 167 µl of isopropanol with muscle glycogen as carrier. The pellet was washed twice with freshly made 70% ethanol. After air-drying, the pellet was resuspended in 11 µl of tris-EDTA pH 8.0 (TE). One microliter of the total RNA sample was applied to a Bioanalyzer to determine the concentration and assay its quality. Ribosomal subtraction was performed using Ribozero kits (Epicentre, Madison, WI) according to the manufacturer’s instructions. The ribosomal depleted RNA was subjected to purification using Agencourt XP beads. The purified RNA was resuspended in 11 µl Rnase free water. Double stranded cDNAs were synthesized from the ribosomal subtracted fractions using the Superscript Double strand cDNA kit (Invitrogen) using random hexamer primers. The amplified cDNAs were diluted to 100 µl and fragmented using Covaris sonication with a duty cycle of 6%, intensity of 4 cycles/bursts of 200 for 320 seconds. The fragmented cDNAs were end-repaired using NEXT repair enzyme mix (New England Biolabs), and A-tailing with Klenow DNA Polymerase. The repaired fragmented cDNAs were ligated to Y-adapters (Illumina). The adapter-ligated products were indexed by PCR amplification using the TruSeq DNA primer set (Illumina). The amplified products were size-fractionated on 6% polyacrylamide gels and a band from 400 to 600 bp was excised. Gel-purified library concentrations were assessed using a Qubit spectrophotometer. Multiplexed libraries were sequenced with Illumina HiSeq technology.

**Unification of embryonic time series samples**

Analyzing expression in the multiple different embryonic time series presents several computational challenges. The starting population of embryos in each of the time series is not homogeneous; instead each contains a distribution of developmental stages, which means the measured expression is actually a convolution of expression values of the individual developmental stages. This initial distribution is expected to persist throughout the growth of the population of embryos. The variance of the distribution of stages can get larger but is not expected to get smaller with time. In addition, the mean developmental time of the initial population of embryos can vary in each experimental time series. As a result the measured clock time of the start of the experimental time series will not exactly correspond to the same developmental time in each experiment. Finally, the growth rates may vary for each different experimental time series, due to subtle differences in growth conditions such as temperature, culture density, and related factors.

To combine the different time series and to estimate more precisely the expression of specific developmental stages, the variation in synchronization and convolution were addressed computationally. The goal computationally is to produce a single unified expression time series based on a standard developmental time scale from the multiple replicate experimental time series. This was accomplished with a Bayesian statistical model. The model relates the measured gene expression to the gene expression of the developmental stages and the proportion of embryos in each stage. The following equation demonstrates this relationship.

$X_{i}= Z * P_{i}+ \epsilon_{i} where i=1...4$

$X_{i}$is the matrix of measured gene expression , the dimension is genes by samples for the *i*th series. Z is the matrix of developmental stage gene expression and has dimension genes by stages. $P_{i}$is the stage distribution for each sample in the *i*th series, and has dimension stages by samples. The entries in this matrix are the fraction of embryos of each stage in the samples. The final term $\epsilon_{i}$is the gaussian error in measurement of the gene expression and has dimension genes by samples. The values of Z and $P_{i}$are the latent variables or parameters of the model.

The dimension of the model is reduced by calculating the values of the P matrices from other parameters. These are 1) the initial mean standard developmental time of the population of embryos, 2) the growth rate of the population of embryos, compared to the standard time scale, 3) the initial distribution of developmental stages in the population, and 4) the increase in the variance of the initial stage distribution over the time of the experiment. These four parameters are inferred for each of the four experimental time series, resulting in 16 parameters estimated from this phase of the unification process. These 16 parameters are used to calculate the proportions entered into the P matrices.

The parameters are inferred from the measured RNA-seq data using the Metropolis-Hastings Markov Chain Monte Carlo (MCMC) algorithm. For this inference we used a subset of 6000 of the most highly expressed genes (>2 dcpm in at least one sample). Genes with values lower than this were considered to have too noisy a signal to contribute to the accurate inference of the parameters. These 16 parameters then allow the synchronization of the time series and the time warping of the sample times.

From these inferred parameters the stage composition of each of the experimental samples was calculated. The stage distribution of a sample is the fraction of embryos in the sample of each developmental stage. With the stage distributions of each sample determined, the final step in the process of unifying the time series is deconvolving to a single time series for the standard developmental stages. This is accomplished with a similar Bayesian model and the Metropolis-Hastings algorithm. The parameters inferred for this second phase model are the gene expression values for each of the 20,000 genes in each of the individual developmental stages. The same deconvolving model can be applied to other features besides genes. Any feature expression measured can be deconvolved once the stage distribution of the samples is determined. In this study the additional features of transcripts, exons, introns, SL1 and SL2 were all deconvolved and unified into single time series by the method. To convert the pseudotime values in standard developmental times, the nuclear counts for the 0 sample in the 0223 series were converted into times after the division into two-cells and the times averaged.


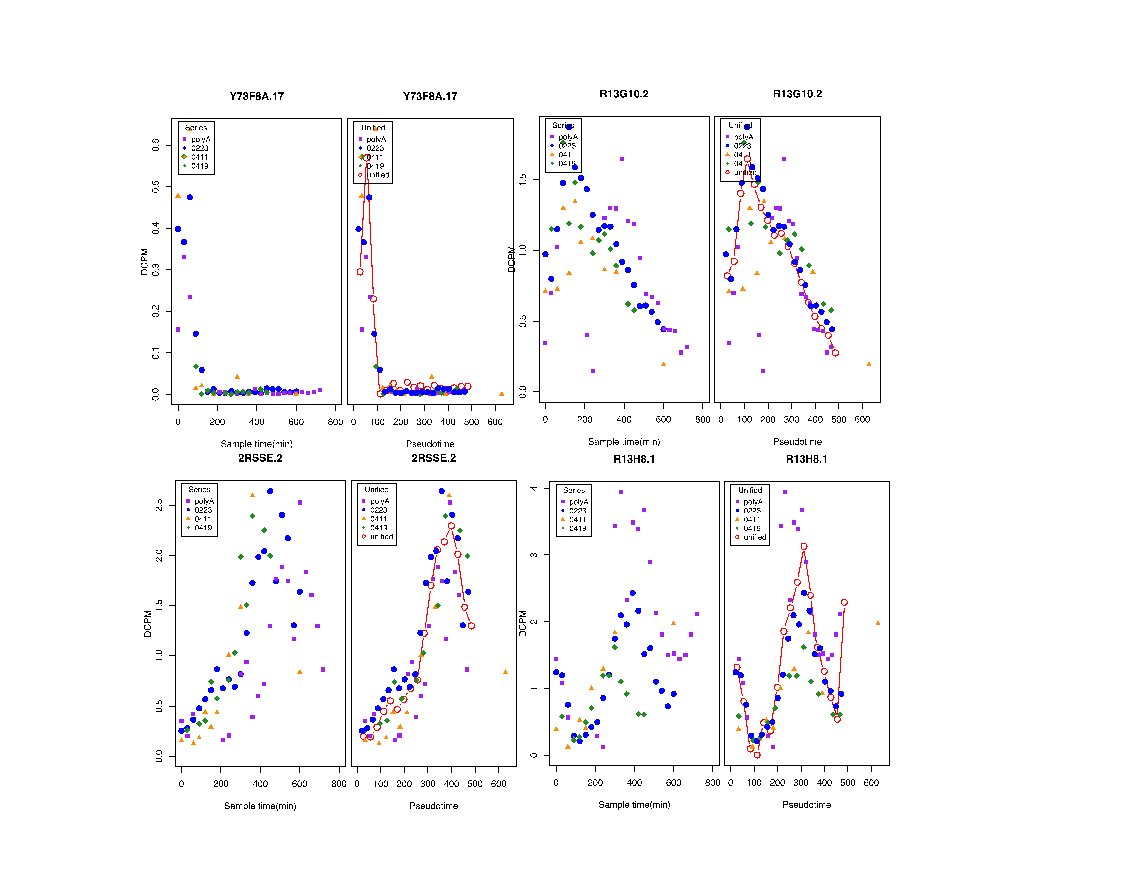


**Methods Figure S1**. Effects of unification. The expression values (dcpm) of the individual samples are plotted for four different genes. In each case the values on the left are plotted according to the time of collection and on the right according to pseudotime based on the 0223 series.

**Alignment/expression quantification**

The RNA-seq alignment process began by identifying all adaptor sequence on the 5’ and 3’ end of the reads, reads beginning with at least 4 Ts (possible polyAs) and reads beginning with at least 6 bases of splice leader (SL) sequence using pattern matching and cross_match (P. Green, unpublished) alignments. Reads were aligned using cross_match against the *C. elegans* genome (WS220; the WormBase sequence remained identical from version WS216 through version WS234) and against a set of *C. elegans* transcript models (Gerstein et al. 2010). If, after integrating the classification and alignment information from the above steps, less than or equal to five bases on either end of the read were unclassified/unaligned, then the read was considered to be mapped. Remaining unmapped reads with at least 30 bases of alignment to the genome were aligned against the genome using a splice aware version of cross_match. If, after integrating the classification from the above steps and splice aware alignment information, less than or equal to five bases on either end of the read were unclassified/unaligned, then the read was considered to be mapped. The remaining unmapped reads were aligned against the *C. elegans* genome using bwasw (Li and Durbin 2010) to identify multi-segment alignments that suggested multiple as yet unpredicted exon pairs. For any multi-segment alignments, the alignment edges were searched to identify splice junctions to join those multi-segment alignments. These alignments were integrated with the initial classification information to determine if the read was to be considered mapped (<=5 bases unaligned from either end).

Final placement for each read was determined by integration of the alignment results from above and defined SLs based on untemplated SLs at the start of the reads and defined polyAs based on untemplated T’s at the start of the read. Putative introns were identified by alignments to the transcriptome and splice aware alignments to the genome. Methods for defining whether those SLs, polyAs, and splice junctions met false positive/false discovery rate thresholds are described (Hillier et al. 2009).

Each transcribed unit was assigned an expression level by its average depth of coverage per base per million reads (dcpm) (Hillier et al. 2009). To distinguish unique regions from repeated sequences, we assigned each base in the genome a representation value as calculated by the number of 24mers from the genome that base participates in, considering all 24mers from both strands. Unique sequence has a representation value of 48, with participation in two unique 24mers, one from each strand at that position, whereas duplicated sequence has a value of 96 or more for more highly repetitive sequence. The average depth of coverage for the transcription unit was determined by summing the coverage of each base for all bases with a representation value of less than 96 divided by the number of bases in the transcript with a representation value of less than 96.  To normalize the results per million reads, the average depth of coverage of the transcript was then multiplied by 1,000,000 and divided by the number of high quality mapped reads (after removing rDNA reads).  In summary, the dcpm is calculated as: s/c/1000000/n , where s = sum of the raw high quality coverage of the bases in that transcript that have a representation value <96, c = number of bases in the transcript that have a representation value < 96, and n = normalized total read coverage of the genome.

The dcpm values were calculated for exons, transcripts and genes. For genes, a dcpm value was calculated by taking a unique list of all of the bases participating in all transcripts for that gene and calculating a single dcpm. For those genes that had no bases with a representation value of 96, the gene family members were identified and the total reads aligning to the gene family were divided equally among each copy and an equal dcpm value was then given to each gene in the family.

**Defining biological replicates**

The tool edgeR (Robinson et al. 2010), used for differential expression analysis, requires biological replicates to find statistically significant differences in gene expression. To find the most closely related samples among our embryo time series data, we calculated Spearman correlations for all sample pairs. We used 6,494 highly transcribed, rapidly changing genes (as defined by a dcpm > 0.4 and a slope of > 6 or < -6 in the change point analysis (see below)). For each sample we identified the top three most similar samples to that sample as defined by their Spearman correlations. We then defined the overall sample order by time using those Spearman data (Methods Table S1). Comparison of the order obtained by the Spearman correlation method to the order obtained using unified sample times reveals a highly similar order.

**Methods Table S1**. Comparison of sample time order by unified time and Spearman correlation

Sample ordered by Unified Samples ordered by

Unified Time Time Spearman correlation

20120223_EMB_0 36 20120223_EMB_0

20120223_EMB_30 69 20120223_EMB_30

20120411_EMB_0 82 20120411_EMB_0

20120419_EMB_30 82 20120419_EMB_30

20120223_EMB_60 102 20120223_EMB_60

20120223_EMB_90 134 20120223_EMB_90

20120411_EMB_60 152 20120411_EMB_60

20120419_EMB_90 160 20120419_EMB_90

20120223_EMB_120 167 20120223_EMB_120

20120411_EMB_90 187 20120411_EMB_-90

20120419_EMB_120 199 20120419_EMB_120

20120223_EMB_150 200 20120223_EMB_150

20120411_EMB_120 222 20120411_EMB_120

20120223_EMB_180 232 20120223_EMB_180

20120419_EMB_150 238 20120419_EMB_150

20120411_EMB_150 257 20120411_EMB_150

20120223_EMB_210 265 20120223_EMB_210

20120419_EMB_180 277 20120419_EMB_180

20120411_EMB_180 292 20120411_EMB_180

20120223_EMB_240 298 20120223_EMB_240

20120223_EMB_270 330 20120223_EMB_270

20120419_EMB_240 355 20120223_EMB_300

20120411_EMB_240 362 20120411_EMB_240

20120223_EMB_300 363 20120419_EMB_240

20120419_EMB_270 394 20120223_EMB_330

20120223_EMB_330 396 20120419_EMB_270

20120223_EMB_360 429 20120223_EMB_360

20120411_EMB_300 433 20120419_EMB_300

20120419_EMB_300 433 20120223_EMB_390

20120223_EMB_390 461 20120411_EMB_300

20120419_EMB_330 472 20120419_EMB_330

20120223_EMB_420 494 20120223_EMB_420

20120411_EMB_360 503 20120419_EMB_360

20120419_EMB_360 511 20120223_EMB_450

20120223_EMB_450 527 20120411_EMB_360

20120223_EMB_480 559 20120223_EMB_480

20120419_EMB_420 589 20120223_EMB_510

20120223_EMB_510 592 20120419_EMB_420

20120223_EMB_540 625 20120419_EMB_450

20120419_EMB_450 628 20120223_EMB_540

20120223_EMB_570 657 20120223_EMB_570

20120223_EMB_600 690 20120223_EMB_600

20120411_EMB_600 784 20120411_EMB_600

We thus defined biological replicates by taking neighboring samples as defined by their Spearman correlations and assigned a time to them using the average time calculated from their unified sample time estimates.

**Methods Table S2**. Average time for sample pairs.

Average

Sample 1 Sample 2 Time

N2_4cell_EE_RZ-56 20120223_EMB_0 28

20120223_EMB_0 20120223_EMB_30 53

20120223_EMB_30 20120411_EMB_0 75

20120411_EMB_0 20120419_EMB_30 82

20120419_EMB_30 20120223_EMB_60 92

20120223_EMB_60 20120223_EMB_90 118

20120223_EMB_90 20120411_EMB_60 143

20120411_EMB_60 20120419_EMB_90 156

20120419_EMB_90 20120223_EMB_120 163

20120223_EMB_120 20120411_EMB_90 177

20120411_EMB_90 20120419_EMB_120 193

20120419_EMB_120 20120223_EMB_150 199

20120223_EMB_150 20120411_EMB_120 211

20120411_EMB_120 20120223_EMB_180 227

20120223_EMB_180 20120419_EMB_150 235

20120419_EMB_150 20120411_EMB_150 247

20120411_EMB_150 20120223_EMB_210 261

20120223_EMB_210 20120419_EMB_180 271

20120419_EMB_180 20120411_EMB_180 285

20120411_EMB_180 20120223_EMB_240 295

20120223_EMB_240 20120223_EMB_270 314

20120223_EMB_270 20120419_EMB_240 343

20120419_EMB_240 20120411_EMB_240 359

20120411_EMB_240 20120223_EMB_300 363

20120223_EMB_300 20120419_EMB_270 378

20120419_EMB_270 20120223_EMB_330 395

20120223_EMB_330 20120223_EMB_360 412

20120223_EMB_360 20120411_EMB_300 431

20120411_EMB_300 20120419_EMB_300 433

20120419_EMB_300 20120223_EMB_390 447

20120223_EMB_390 20120419_EMB_330 467

20120419_EMB_330 20120223_EMB_420 483

20120223_EMB_420 20120411_EMB_360 498

20120411_EMB_360 20120419_EMB_360 507

20120419_EMB_360 20120223_EMB_450 519

20120223_EMB_450 20120223_EMB_480 543

20120223_EMB_480 20120419_EMB_420 574

20120419_EMB_420 20120223_EMB_510 590

20120223_EMB_510 20120223_EMB_540 608

20120223_EMB_540 20120419_EMB_450 626

20120419_EMB_450 20120223_EMB_570 643

20120223_EMB_570 20120223_EMB_600 674

20120223_EMB_600 20120411_EMB_600 737

We can then identify biological replicate pairs that are separated by equal intervals.

**Methods Table S3**. Average time for biological replicate pairs separated by 80 minutes (same table is obtained when using 60 minutes).

Average

Sample 1 Sample 2 Time

N2_4cell_EE_RZ-56 20120223_EMB_0 28

20120223_EMB_60 20120223_EMB_90 118

20120419_EMB_120 20120223_EMB_150 199

20120419_EMB_180 20120411_EMB_180 285

20120223_EMB_300 20120419_EMB_270 378

20120223_EMB_390 20120419_EMB_330 467

20120223_EMB_480 20120419_EMB_420 574

20120223_EMB_570 20120223_EMB_600 674

100 minutes

N2_4cell_EE_RZ-56 20120223_EMB_0 28

20120223_EMB_90 20120411_EMB_60 143

20120419_EMB_150 20120411_EMB_150 247

20120419_EMB_240 20120411_EMB_240 359

20120223_EMB_390 20120419_EMB_330 467

20120223_EMB_480 20120419_EMB_420 574

20120223_EMB_570 20120223_EMB_600 674

120 minutes

N2_4cell_EE_RZ-56 20120223_EMB_0 28

20120411_EMB_60 20120419_EMB_90 156

20120419_EMB_180 20120411_EMB_180 285

20120223_EMB_330 20120223_EMB_360 412

20120223_EMB_450 20120223_EMB_480 543

20120223_EMB_570 20120223_EMB_600 674

Using these biological sample replicate pairs and looking at a defined time interval between samples (e.g. 80 minutes, 100 minutes, 120 minutes), we then identified the genes that were up and down regulated in each time interval using edgeR (Robinson et al. 2010) as detailed below.

**Differential expression analysis using edgeR**

Read counts per gene were used as input to edgeR to identify those genes that were up and down regulated between developmental stages. In each case, biological replicate pairs (as defined by the Spearman correlation analysis described above) were used. For each comparison we only included the genes that had a dcpm of at least 0.07 in at least one of the samples used to increase the statistical power of the analysis of differential expression (Anders et al. 2013). We examined both the specific genes as well as their GO categories.

**GO analysis**

For each set of up/down regulated genes from the unified time series, from the edgeR comparison and from the group of maximally expressed genes, the genes were examined for enrichment of gene ontology (GO) terms using the online GO database GOminer (Zeeberg et al. 2003). Standard parameters were used to uncover enrichments. Biological, cellular and molecular databases for ontological terms were searched. Terms were then clustered using Ward minimum variance hierarchical clustering based on enrichment for each gene set and plotted using R. For the ratio plots the number of genes in each maximally expressed time point that overlapped with those genes in each of the up or down regulated time points was calculated. The overlap was divided by the number of maximally expressed genes for that time point. The values were then plotted using R and ggplot (Wickham 2009; R Core Team 2015).

**Change point analysis**

A Bayesian statistical model was used to detect change points in the unified embryonic gene expression time series. A reversible jump Monte Carlo Markov Chain (MCMC) algorithm (Green 1995) infers the number and location of the change points in developmental time. For each gene the number of change points was limited to less than three. The generative model assumes that the time series expression is a linear function of time and the slope of that function changes at the change points. This model results in a piece-wise linear function, representing the unified expression time series for each gene measured, with one to four possible segments. To prevent over-fitting, an exponential distribution was used as the prior on the number of change points. The effect is to decrease the probability of adding an additional change point. Without the prior, the model would result in choosing the maximum number of change points allowed for each gene. The piece-wise linear approximation of the time series facilitates downstream computational analysis.


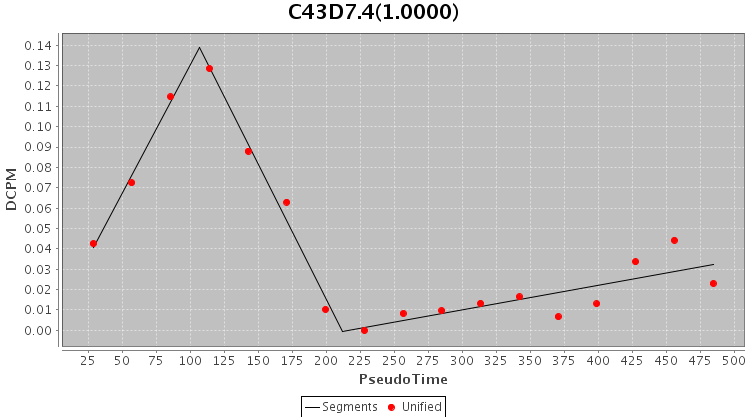

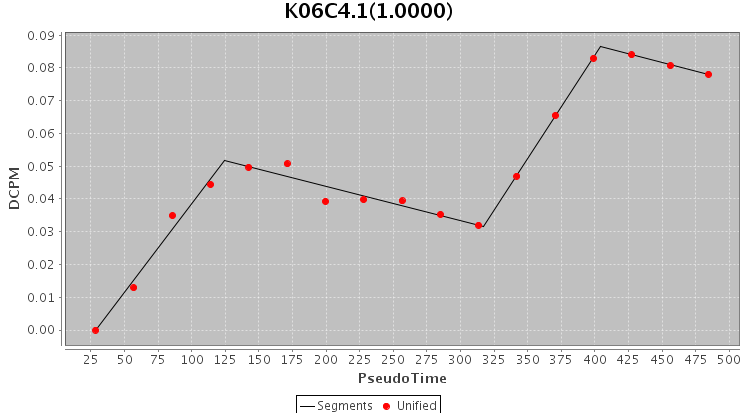

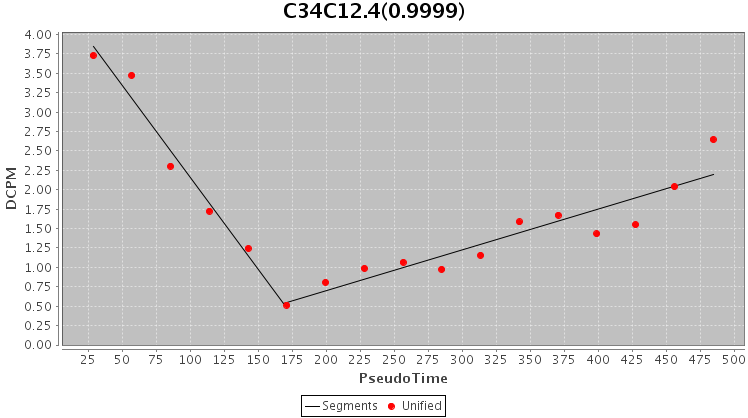

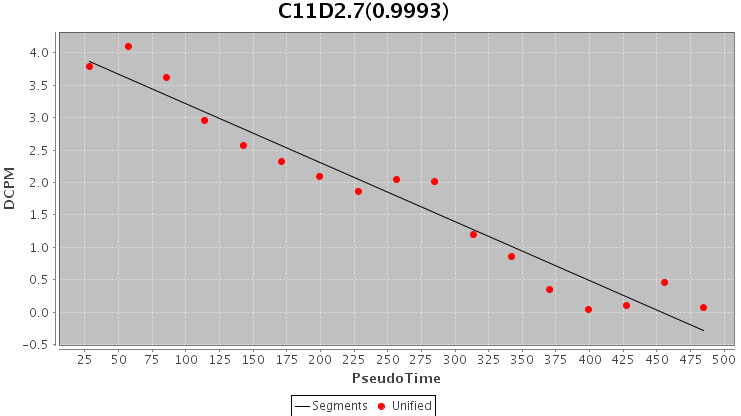


**Methods Figure S2**. Examples of change point analysis. Expression patterns with three, four, two and one segments are shown. The examples span a range of expression values.

**Differential intron usage**

In order to find introns that are alternatively spliced and used differentially during the life cycle, we first identified all junction sites that linked to two or more other sites (single donor site linking to two or more acceptor sites or vice versa). To minimize artifacts we only considered junctions where the total number of reads spanning the junction summed across all samples was greater than 10 reads and greater than 1% of the other junctions in that gene. The junction with the most reads was designated the major form and the other(s) designated the minor form(s). Each of the minor form was then considered pairwise against the major form. Only minor forms that were at least 1% of the major form were used, producing a total of 7279 alternatively spliced pairs. To compare expression of the minor and major forms across the life cycle, we calculated the fraction of total reads for the minor form in each sample and similarly for the major form, thus normalizing for the different levels of expression of the two forms. Using the normalized expression fractions, we calculated the ratio of the minor form to the total of both forms. The distribution of ratios for all junctions across all samples indicated that frequency of ratios greater than 0.85 was less than 7% and the frequency of ratios less than 0.15 was less than 40%. The asymmetry resulted from the large number of samples in which the minor form was not expressed at all. Finally, we looked for consecutive samples (runs), allowing up to two, exceptions, in which the ratio exceeded 0.85 or was less than 0.15. Also, at least one of the forms had to have expression of at least 0.1 of the maximum expression for that form. This latter filter avoided samples in which overall expression was very low. In Suppl. Table S8, we report the two longest runs for each intron pair, the gene containing that pair, and the stage at which the run begins. We report the results separately for instances in which the minor form predominates (ratio > 0.85) and in which the major form predominates (ratio < 0.15). Graphs for each pair of junctions were generated, showing in one graph the relative expression of each member of the pair across the life cycle and in a second the ratio of the minor form to the total across the life cycle (<http://genome.sfu.ca/gexplore/gexplore_search_expression.html>).

**Operons**

Operon annotation was obtained from WormBase build WS220. The ratio of SL2 over total SL dcpm, SL2/(SL1+SL2), was calculated for each gene at each time point in every operon for the samples of the 0223 series. Operons were then ranked based on this ratio for the second gene in the operon averaged across the first 7 time points or 3 hours of development. These time points were chosen since they corresponded most closely to the developmental stage that the chromatin data was obtained from (Liu et al. 2011). To make the analysis more robust, we excluded all operons whose second gene was expressed at an average of less than 0.1 dcpm across all stages. Of an initial 913 total operons, 840 operons passed this threshold. Operons were then placed in 8 bins consisting of 105 genes each for further analysis. For each bin of 105 operons we calculated 1) the average of the SL2 ratio for first and second gene, average SL2 for first and second genes, average SL1 for first and second, 2) the average distance of second gene TSS to the first gene polyA, 3) the average correlation of expression pattern between the first and second gene and 4) first gene expression for each bin of 105 operons. We also mined existing chromatin datasets, calculating the average signal surrounding the TSS of both first and second genes. For each bin we plotted the average signal 1000 bp either side of the TSS.

**Non coding RNA**

To find all the annotated coding and ncRNA transcripts, all of the following Wormbase (WS245) categories were included: Coding_transcript, Non_coding_transcript, Pseudogene, RNASEQ.Hillier, RNASEQ.Hillier.Aggregate, Transposon_CDS, Transposon_Pseudogene, asRNA, curated, lincRNA, miRNA_mature, miRNA_precursor, ncRNA, piRNA, rRNA, scRNA, snRNA, snoRNA, and tRNA. All WS245 coordinates were converted to WS225 coordinates for the analyses presented here. All aggregate transcripts from a *C. elegans* predicted transcript set (Gerstein et al., 2014) were also included to define additional exons. Introns were as defined by WormBase annotation.

All exons and introns from the above sets were masked and the remaining bases were defined as “intergenic.” All exons from the ncRNA categories (asRNA, curated, lincRNA, miRNA_mature, miRNA_precursor, ncRNA, piRNA, rRNA, scRNA, snRNA, snoRNA, tRNA) that did not overlap coding exons were used to define the ncRNA category and divided into those that were less than 100 bases and those that were greater than or equal to 100 bases.

**Methods Table S4**. Number of bases per feature category.

| Category | Mb |
| --- | --- |
| coding exon | 28.38 |
| intergenic | 30.84 |
| intronic | 34.22 |
| ncRNA >=100 intergenic | 0.71 |
| ncRNA <100 intergenic | 0.42 |
| ncRNA >= 100 intronic | 0.34 |
| ncRNA < 100 intronic | 0.16 |

The coverage of each base in the genome was calculated using only the high quality/uniquely mapping reads. The raw read coverage value was normalized by obtaining the raw reads per million bases at each base (multiplying the number of reads aligning to each base by 1,000,000 and dividing by the number of reads mapped in that sample, where the number of reads mapped in that sample is normalized to assume that all reads in all samples are 35 bases). The normalized coverage of each base of each of the categories was analyzed on a per chromosome/per sample basis. Only samples with a 5% intergenic ROC threshold of <=200 were included (totalRNA: 20120223_EMB-0, 20120223_EMB-30, 20120223_EMB-60, 20120411_EMB-0, 20120411_EMB-120, 20120411_EMB-60, 20120419_EMB-120, 20120419_EMB-30; polyA+ N2_EE_50-0,N2_EE_50-210,N2_EE_50-240,N2_EE_50-30,N2_EE_50-330,N2_EE_50-360,N2_EE_50-390,N2_EE_50-420,N2_EE_50-450,N2_EE_50-480,N2_EE_50-510,N2_EE_50-540,N2_EE_50-570,N2_EE_50-60,N2_EE_50-600,N2_EE_50-630,N2_EE_50-660,N2_EE_50-690,N2_EE_50-720).

**Potential transcribed bases not annotated in WormBase**

To look for possible transcription outside of annotated transcripts, first, all WormBase (WS245 annotation mapped to WS225 coordinates) annotated exons (and adding 50 bases in each direction to the prediction) including those in pseudogenes, ncRNAs, coding and non-coding transcripts were masked. Second, all bases in our aggregate transcript set were masked. Then, all bases whose aggregate coverage exceeded the coverage below (where the coverage is a windowed coverage summing raw high quality coverage across the 25 bases to the left and the right of the base under consideration) were identified. We counted blocks of above threshold coverage that were not contiguous with WormBase exons for both the aggregate projects and only the polyA+ projects (Suppl. Table S14). For example, at a threshold of 1500 (the threshold used for creating transcript predictions in the aggregate set), there are 12,861 blocks of longer than 100 bases and 101 blocks that are 2000 bases or longer that are not immediately adjacent to a WormBase prediction.

**Pervasive transcription**

Each chromosome was divided into non-overlapping 80 Kbase regions. The bases were annotated as intergenic, coding, or non-coding. Per base dcpm is arithmetically averaged over the bases of the regions for each type of annotation for each the high quality embryonic samples. The regional dcpm values are then arithmetically averaged over the samples. This produces a single vector of dcpm values for each chromosome and each annotation type. These vectors are then compared by calculating the Spearman correlation. This comparison was done with each pair of annotated features. To assess the value of the random correlation between the pairs of annotated features, the intergenic vector is randomly permuted 1000 times and the permuted intergenic vector is correlated with the coding vector. The mean and the standard deviation of the 1000 Spearman correlation values is calculated. This is used to calculate the standard Z-score of the Spearman correlation values obtained from the non-randomized intergenic vector and the coding vector. A graphic representation of the correlation between the annotation pairs is provided. Since the distribution of regional dcpm values is found to be exponential (not Gaussian) across the chromosomes, the geometric standard score is used to compare the dcpm values of the different annotation types. The geometric standard score of the regional dcpm values is plotted against the genomic position in the chromosome. When calculating the Spearman correlation between two types of features, any region that does not have bases annotated with both the types is excluded from the Spearman correlation calculation.

**References for Methods**

Anders S, McCarthy DJ, Chen Y, Okoniewski M, Smyth GK, Huber W, Robinson MD. 2013. Count-based differential expression analysis of RNA sequencing data using R and Bioconductor. *Nat Protoc* **8**(9): 1765-1786.

Boyle TJ, Bao Z, Murray JI, Araya CL, Waterston RH. 2006. AceTree: a tool for visual analysis of *Caenorhabditis elegans* embryogenesis. *BMC Bioinformatics* **7**: 275.

Gerstein MB Lu ZJ Van Nostrand EL Cheng C Arshinoff BI Liu T Yip KY Robilotto R Rechtsteiner A Ikegami K et al. 2010. Integrative analysis of the *Caenorhabditis elegans* genome by the modENCODE project. *Science* **330**(6012): 1775-1787.

Green PJ. 1995. Reversible jump Markov chain Monte Carlo computationand Bayesian model determination. *Biometrika* **82**: 711–732.

Hillier LW, Reinke V, Green P, Hirst M, Marra MA, Waterston RH. 2009. Massively parallel sequencing of the polyadenylated transcriptome of *C. elegans.* *Genome Res* **19**(4): 657-666.

Li H, Durbin R. 2010. Fast and accurate long-read alignment with Burrows-Wheeler transform. *Bioinformatics* **26**(5): 589-595.

Liu T, Rechtsteiner A, Egelhofer TA, Vielle A, Latorre I, Cheung MS, Ercan S, Ikegami K, Jensen M, Kolasinska-Zwierz P et al. 2011. Broad chromosomal domains of histone modification patterns in *C. elegans*. *Genome Res* **21**(2): 227-236.

R Core Team. 2015. A language and environment for statistical computing. R Foundation for Statistical Computing, Vienna, Austria.

Robinson MD, McCarthy DJ, Smyth GK. 2010. edgeR: a Bioconductor package for differential expression analysis of digital gene expression data. *Bioinformatics* **26**(1): 139-140.

Wickham H. 2009. *ggplot2: elegant graphics for data analysis*. Springer, New York.

Zeeberg BR, Feng W, Wang G, Wang MD, Fojo AT, Sunshine M, Narasimhan S, Kane DW, Reinhold WC, Lababidi S et al. 2003. GoMiner: a resource for biological interpretation of genomic and proteomic data. *Genome Biol* **4**(4): R28.
