# Supplementary material for: The time-resolved transcriptome of C. elegans
Source: Genome Res. 2016 Oct;26(10):1441–50. doi: 10.1101/gr.202663.115 (PMC5052054; doi:10.1101/gr.202663.115)
Supplement: Supplemental Material [file supp_gr.202663.115_Supplemental_Table_S7.docx]

**Supplemental Table 7:** Change points of transcription factors in regulatory cascades

| Gene name | Common name | unification stage | Embryo time (min) | initial slope | following slope |
| --- | --- | --- | --- | --- | --- |
| T24D3.1 | *med-1* | 2.14 | 89 | 4.97 | -18.16 |
| F58E10.5 | *end-3* | 2.17 | 90 | 12.27 | -13.43 |
| F58E10.2 | *end-1* | 3.26 | 132 | 7.71 | -8.27 |
| C18G1.2 | *elt-7* | 4.82 | 193 | 8.42 | -5.78 |
| C33D3.1 | *elt-2* | 7.3 | 290 | 6.15 | -1.94 |
|  |  |  |  |  |  |
| ZK177.10 | *tbx-35* | 2.89 | 118 | 17.23 | -33.07 |
| C38D4.6 | *pal-1* | 3 | 122 | -11.52 | -0.85 |
| C44C10.8 | *hnd-1* | 4.56 | 183 | 10.02 | -8.02 |
| B0304.1 | *hlh-1* | 5.34 | 213 | 8.54 | -2.24 |
| D1081.2 | *unc-120* | 6.89 | 274 | 5.07 | 1.3 |
