# Supplementary material for: The time-resolved transcriptome of C. elegans
Source: Genome Res. 2016 Oct;26(10):1441–50. doi: 10.1101/gr.202663.115 (PMC5052054; doi:10.1101/gr.202663.115)
Supplement: Supplemental Material [file supp_gr.202663.115_Supplemental_Fig_S1.docx]

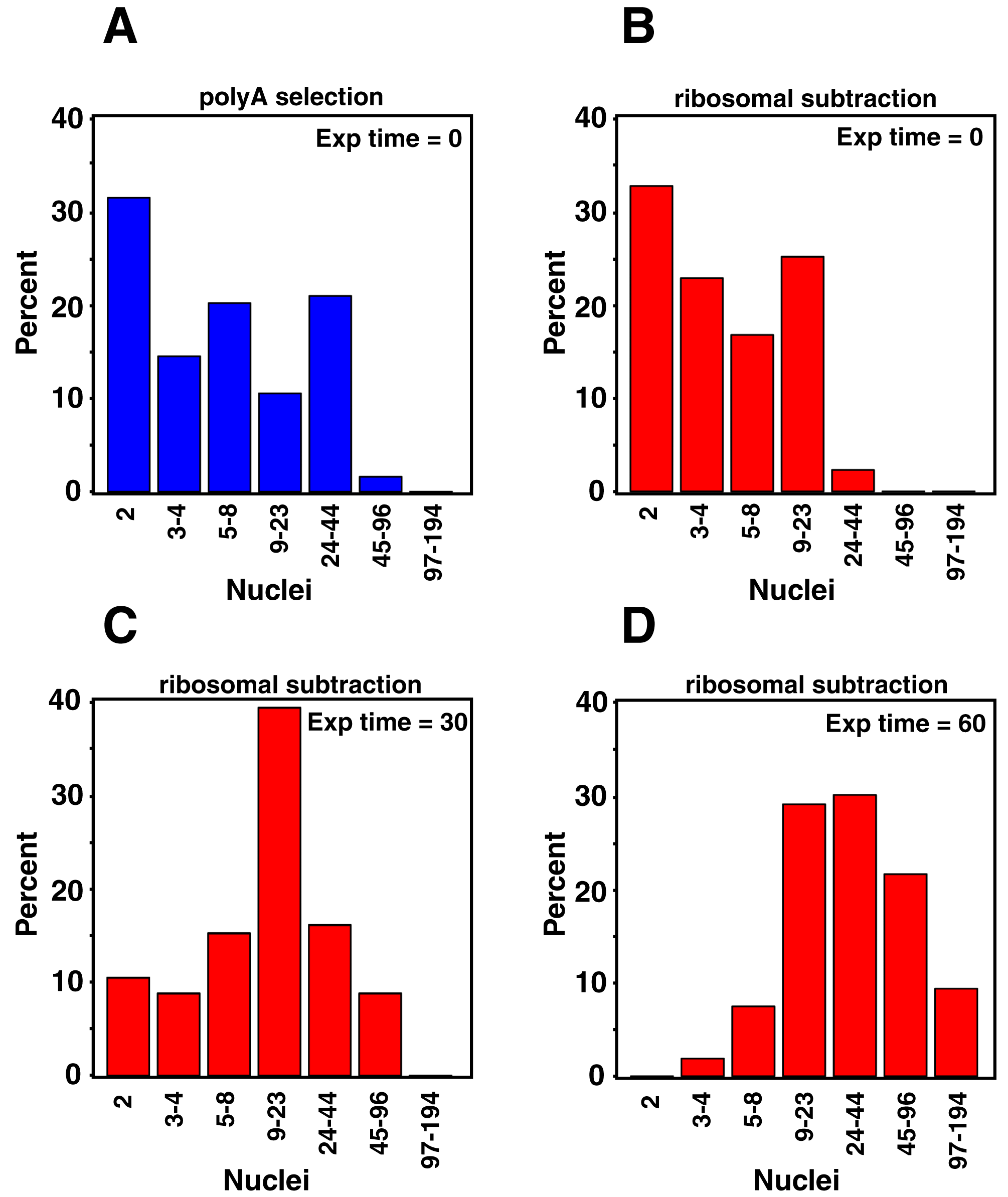


Supplemental Figure 1. Nuclear counts per sample. A) Nuclear counts of the initial sample in the times series using polyA selection. B) Nuclear counts of the initial sample of the 0223 series, using ribosomal rRNA subtraction. C) and D) Nuclear counts of samples 30 and 60 minutes after the initial time point. The initial sample in the polyA selected sample has a slightly broader distribution of embryo stages than the initial sample in the 0223 series. With later time samples in the 0223 series the distribution shifts to the right (older embryos) as expected. See methods for details of counting procedures.
