# Supplementary material for: The time-resolved transcriptome of C. elegans
Source: Genome Res. 2016 Oct;26(10):1441–50. doi: 10.1101/gr.202663.115 (PMC5052054; doi:10.1101/gr.202663.115)
Supplement: Supplemental Material [file supp_gr.202663.115_Supplemental_Fig_S2.docx]

Supplemental Figure 2. Relationship between the samples of the four time series and the unified values. The inferred times of each of the samples from the polyA selected series, the three rRNA subtracted series and the unified set are plotted relative to one another. The relative positions are derived from the Bayesian analysis of the RNA-seq data. The different series were then scaled to embryonic time points based on the nuclear counts of the initial 0223 series samples and the knowledge that subsequent samples were collected every 30 minutes. Overall, the 63 different samples are well scattered over most of embryonic development, but very late embryogenesis is undersampled. The imputed time points for samples in each series in minutes post-2-cell are as follows:


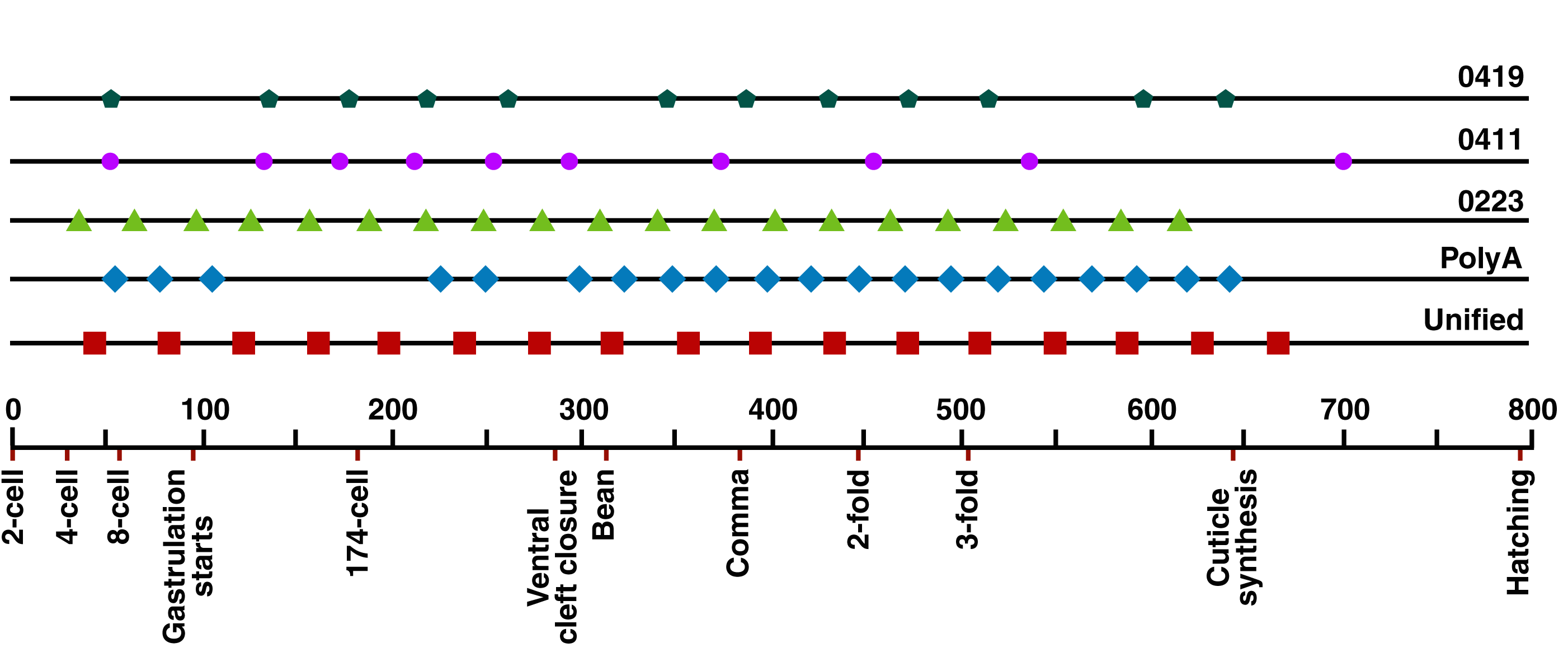


**Unified series** (44, 83, 122, 161, 199, 238, 277, 316, 355, 393, 432, 471, 510, 548, 587, 626, 665).

**PolyA series** (54, 78, 103, 225, 249, 298, 323, 347, 371, 396, 420, 445, 469, 493, 518, 542, 567, 591, 616, 640).

**0223 series** (35, 65, 96, 126, 157, 187, 218, 248, 279, 309, 340, 370, 401, 431, 462, 492, 523, 553, 584, 614, 645).

**0411 series** (52, 133, 173, 213, 254, 294, 374, 455, 536, 700).

**0419 series** (53, 136, 178, 220, 262, 346, 388, 430, 472, 514, 597, 639).
