# Supplementary material for: The time-resolved transcriptome of C. elegans
Source: Genome Res. 2016 Oct;26(10):1441–50. doi: 10.1101/gr.202663.115 (PMC5052054; doi:10.1101/gr.202663.115)

Supplemental Figure 3. Correlation of time series with single embryo data (Hashimshony et al. 2015). A) The Spearman correlations of the single embryo data obtained with Cel-seq with the synchronized bulk samples of the 0223 rRNA subtracted time series. Correlations within the two different series appear along the diagonal and correlations between the series appear at the upper right and lower left quadrants. Maximal correlation of samples between the series centers around equivalent times in both series. The correlations between nearby time points fall off substantially after 480 minutes both within the single embryo data and between that and the 0223 series. In contrast, the correlations between nearby later time points in the 0223 remain high. The source of this discrepancy is unclear. B) Spearman correlations of the single embryo data with the unified stages derived by Bayesian analysis of all four bulk time series, arranged as above. The correlations between nearby stages fall off more rapidly in the unified values than in the 0223 series, and the overall shape more closely resembles that of the single embryo series. Both differences show that the unified values have successfully deconvolved the different stages in the bulk samples of the 0223 series. Again the correlations of the unified values with the single embryo data fall off sharply at later time points.


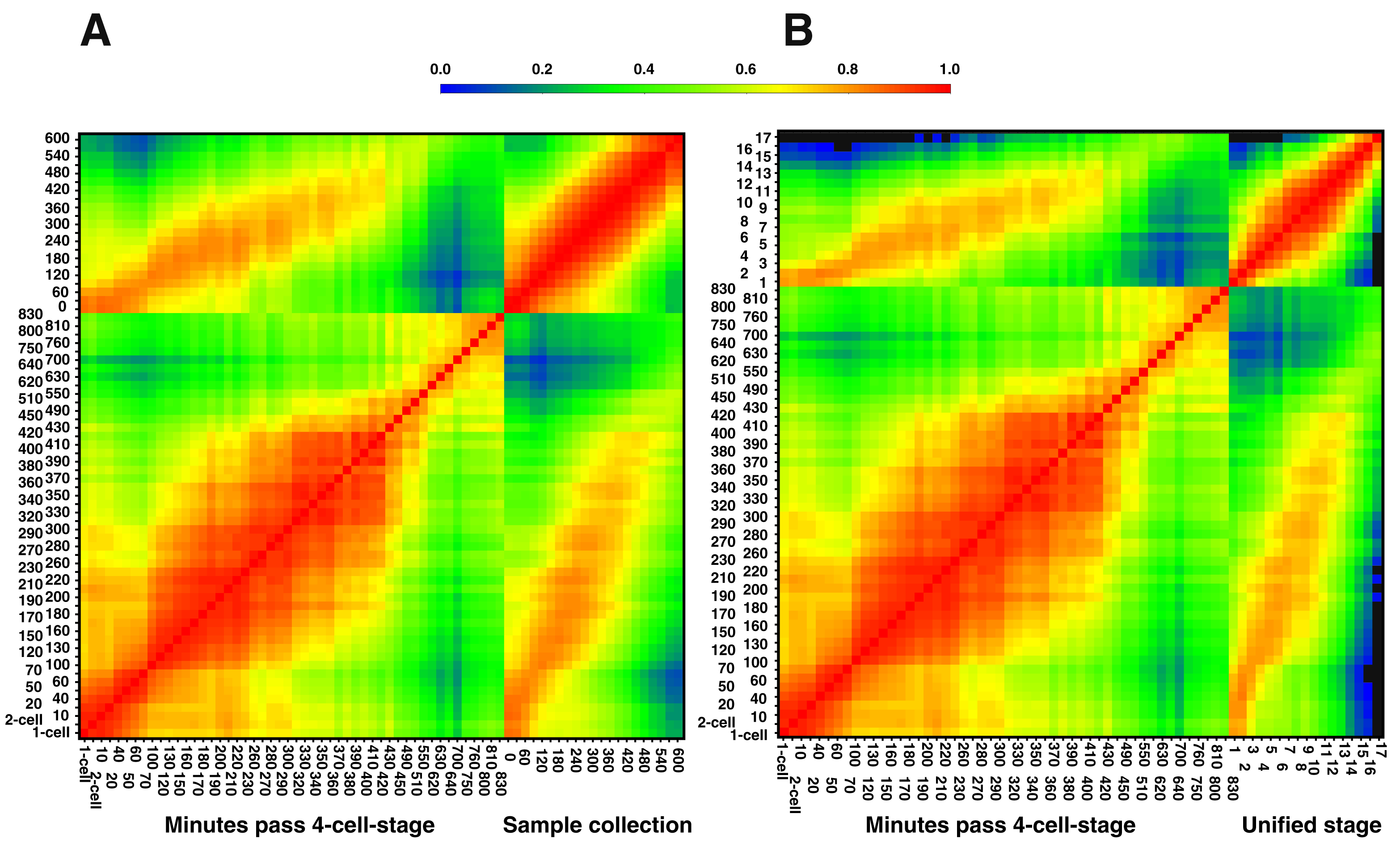

Supplement: Supplemental Material [file supp_gr.202663.115_Supplemental_Fig_S3.docx]
