# Supplementary material for: The time-resolved transcriptome of C. elegans
Source: Genome Res. 2016 Oct;26(10):1441–50. doi: 10.1101/gr.202663.115 (PMC5052054; doi:10.1101/gr.202663.115)
Supplement: Supplemental Material [file supp_gr.202663.115_Supplemental_Fig_S4.docx]

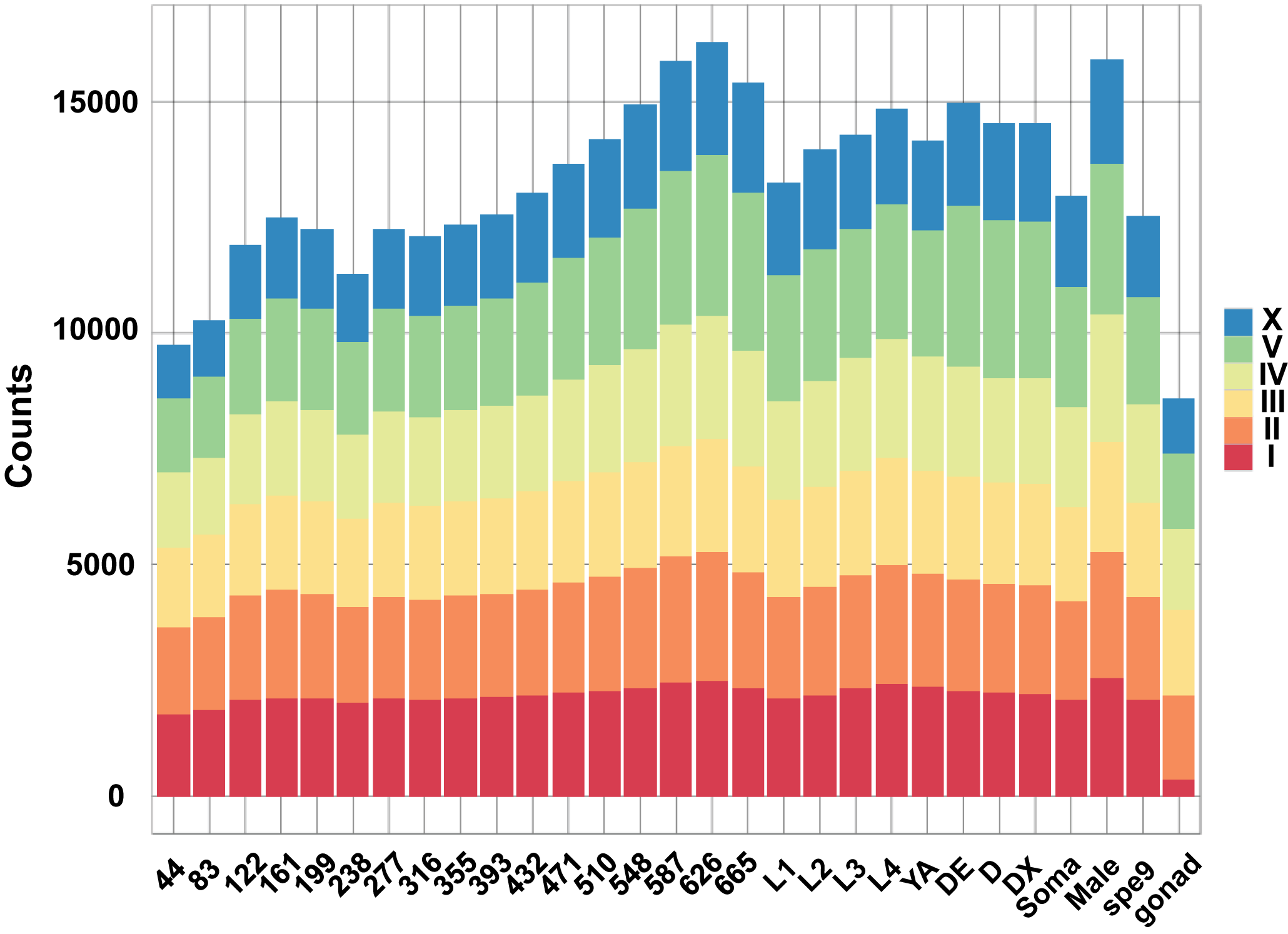


Supplemental Figure 4. Total genes with at least 0.02 dcpm at each stage. For each stage the total number of genes expressed above 0.02 was counted for each chromosome. As expected the total number of genes expressed over this threshold increased through embryogenesis as the complexity of the embryo increased. By the penultimate embryonic stage nearly three quarters of all genes were expressed above 0.02 dcpm. The number of genes above this threshold dropped during the larval and adult stages and was at its lowest for the tissue-specific samples, indicating that this threshold is an indication of the level of complexity of a sample. Interestingly, the time points with the highest number of expressed genes are the late embryo and the L4 male sample.
