# Supplementary material for: The time-resolved transcriptome of C. elegans
Source: Genome Res. 2016 Oct;26(10):1441–50. doi: 10.1101/gr.202663.115 (PMC5052054; doi:10.1101/gr.202663.115)
Supplement: Supplemental Material [file supp_gr.202663.115_Supplemental_Fig_S5.docx]

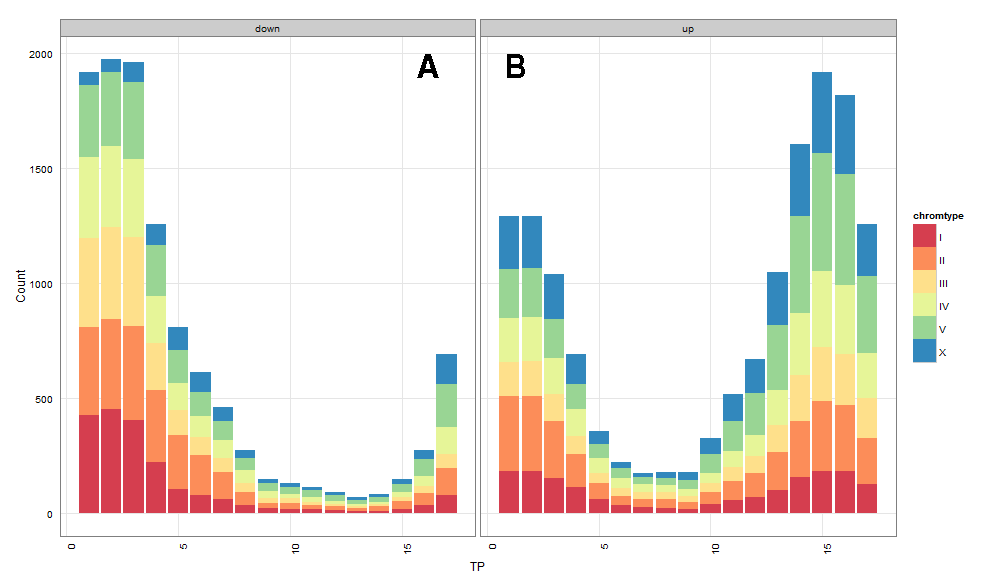

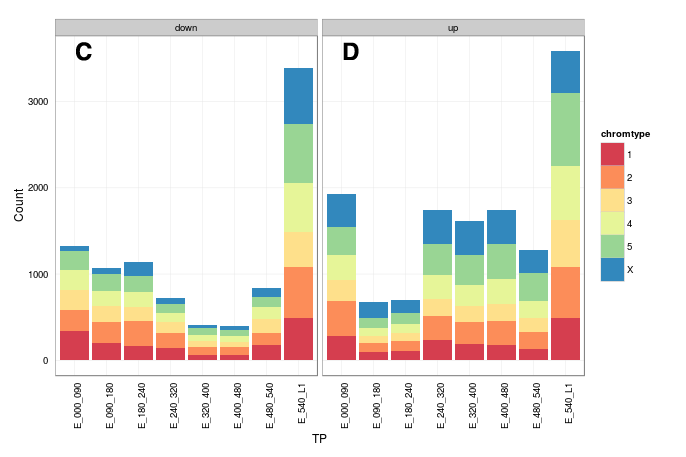


Supplemental Figure 5. Genes increasing and decreasing in expression at each stage by change-point and edgeR analysis. A) Genes decreasing in expression by stage. Change point analysis was used to determine the rate of change of each gene over the course of embryogenesis (see methods for details). The resulting file was parsed to yield a rate of change for each time point in the unified series. In turn the number of genes with a normalized slope of less than 7 and a maximum expression of greater than 0.1 dcpm were counted for each stage and for each chromosome and plotted. The genes decreasing in expression at the first time point are almost entirely maternally derived mRNAs, based on having maximal expression at the first embryonic time point, their increase from the L4 to the young adult stage and their abundance in the dissected gonad. As expected these genes are underrepresented on the X chromosome. By the third stage already 15% of the decreasing mRNAs represent early zygotic genes that have already reached their peak and are decreasing. B) Genes increasing in expression by stage. Those genes increasing at the first time point are early zygotically expressed genes. Their overall numbers is smaller than the set of genes increasing late in embryogenesis. The number of genes increasing in expression during the middle stages of embryogenesis is relatively few (see main text for further discussion). C) Genes down-regulated using edgeR and D) up-regulated according to edgeR.
