# Supplementary material for: The time-resolved transcriptome of C. elegans
Source: Genome Res. 2016 Oct;26(10):1441–50. doi: 10.1101/gr.202663.115 (PMC5052054; doi:10.1101/gr.202663.115)

Supplemental Figure 6. Expression patterns of gene families.


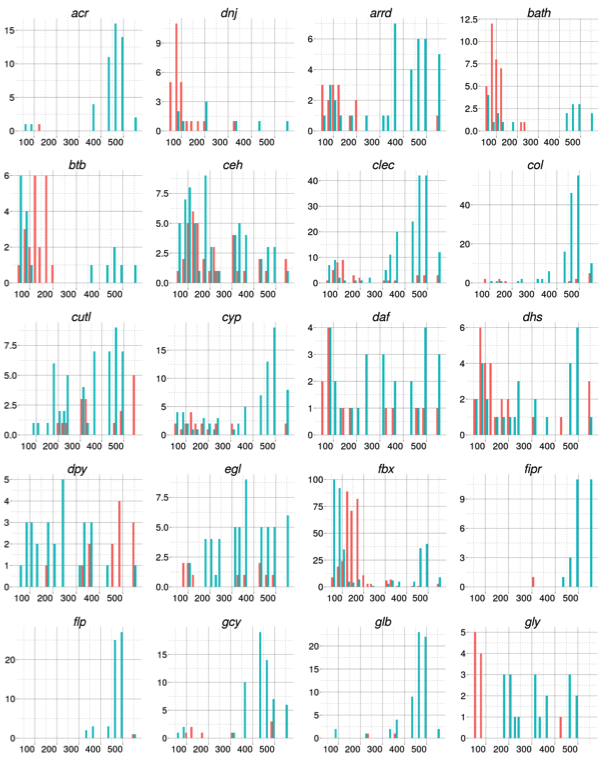


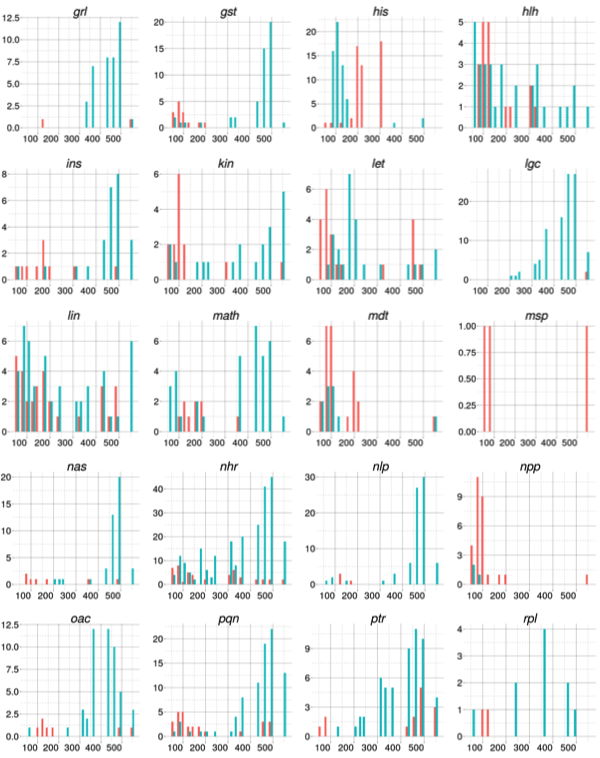


Supplemental Figure 6. Expression patterns of gene families. For common gene names (top) with 20 or more members, the number of up- (green) and down- (red) regulated genes between adjacent stages is shown. The numbers on the x-axis refer to the minutes beyond the four cell stage (inferred from the unification analysis of the later of the two stages being compared and the y-axis gives the number of genes changing in that interval. Similar trends were found using edgeR. The latter also allowed us to examine late embryo stages against the L1 (not shown).


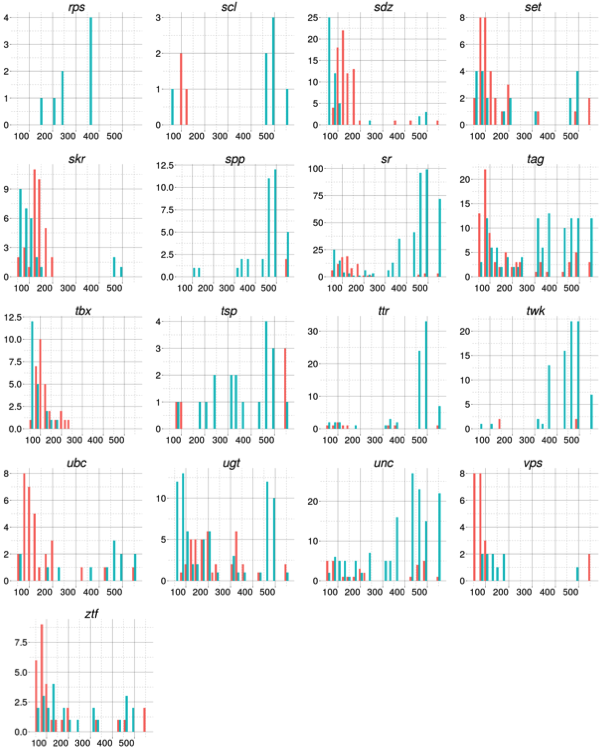

Supplement: Supplemental Material [file supp_gr.202663.115_Supplemental_Fig_S6.docx]
