# Supplementary material for: The time-resolved transcriptome of C. elegans
Source: Genome Res. 2016 Oct;26(10):1441–50. doi: 10.1101/gr.202663.115 (PMC5052054; doi:10.1101/gr.202663.115)
Supplement: Supplemental Material [file supp_gr.202663.115_Supplemental_Fig_S7.docx]

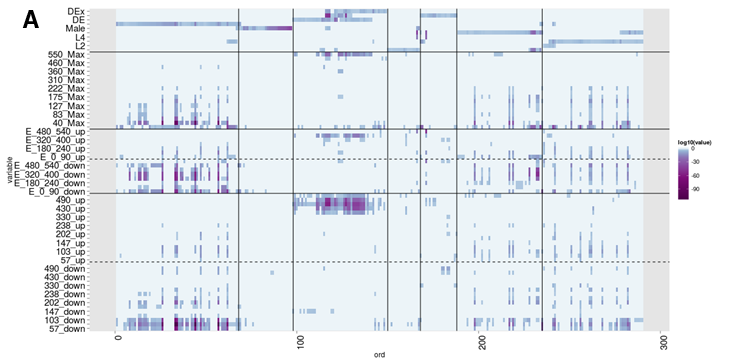


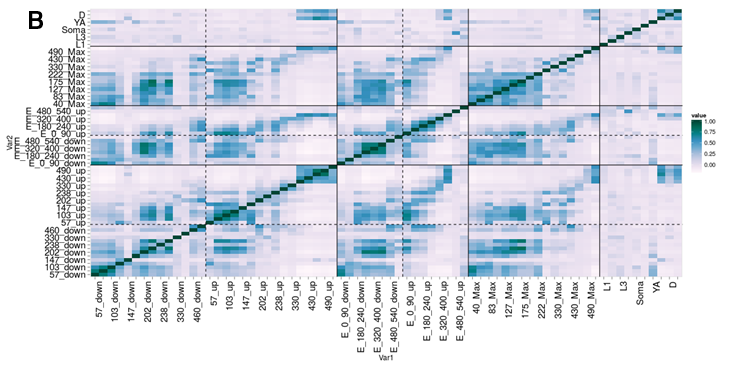


Supplemental Figure 7 - GO analysis. A) We determined the GO enrichment for all the genes found to be up- and down-regulated at each time point, each edgeR annotated comparison and each set of maximally expressed genes. We compiled the enrichment for 2,376 terms that showed enrichment at least a single time point for all these stages and comparisons. We then clustered the enrichment values based on the adult-stage p-values while examining the shared values with the embryonic time points. We found that there was significant overlap of terms between the young adult stages (first block) and those terms down-regulated early in development and maximally expressed early, between the dauer stages (third block) and those terms up-regulated at the end of embryogenesis. B) We then calculated the Spearman correlation for the GO term enrichment for each time point and comparisons. There was a high correlation between those terms up-regulated late in development and the dauer stages. Interestingly there is very little correlation between those later time points and any stage other than the dauer stages.
