# Supplementary material for: The time-resolved transcriptome of C. elegans
Source: Genome Res. 2016 Oct;26(10):1441–50. doi: 10.1101/gr.202663.115 (PMC5052054; doi:10.1101/gr.202663.115)

Supplemental Figure 8. Venn diagram of the overlap of detected splice junctions with WormBase junctions. In toto our data sets revealed 171,827 splice junctions that had expression levels of at least 1% of the average level for junctions in that gene and were detected in more than one sample. Only 474 of confirmed WormBase junctions are not represented in our set and we detect 61,728 junctions not represented in WormBase.


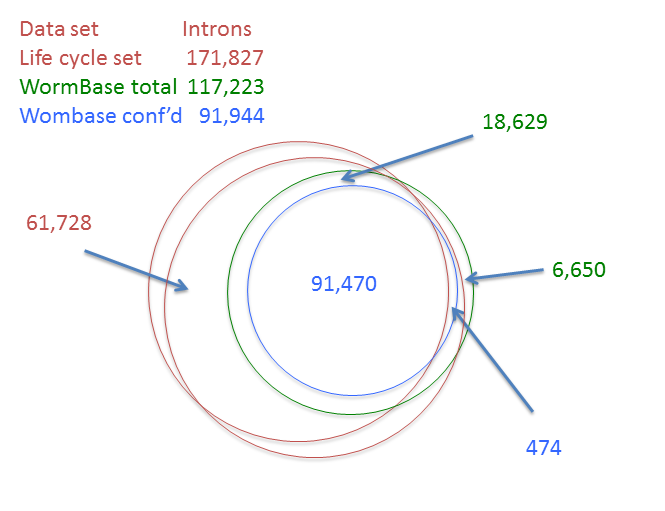

Supplement: Supplemental Material [file supp_gr.202663.115_Supplemental_Fig_S8.docx]
