# Supplementary material for: The time-resolved transcriptome of C. elegans
Source: Genome Res. 2016 Oct;26(10):1441–50. doi: 10.1101/gr.202663.115 (PMC5052054; doi:10.1101/gr.202663.115)
Supplement: Supplemental Material [file supp_gr.202663.115_Supplemental_Fig_S9.docx]

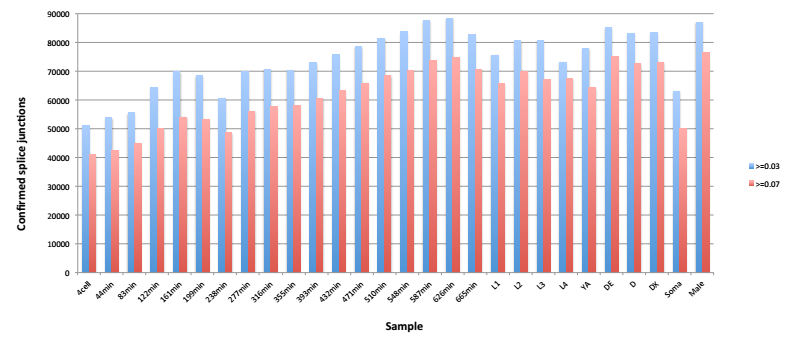


Supplemental Figure 9. The number of splice junctions confirmed in each sample at a dcpm threshold of 0.03 and 0.07 are shown. There are a total of 157,292 splice junctions confirmed across these samples at >=0.03 and 143,054 at >=0.07.
