# Supplementary material for: The time-resolved transcriptome of C. elegans
Source: Genome Res. 2016 Oct;26(10):1441–50. doi: 10.1101/gr.202663.115 (PMC5052054; doi:10.1101/gr.202663.115)
Supplement: Supplemental Material [file supp_gr.202663.115_Supplemental_Fig_S10.docx]

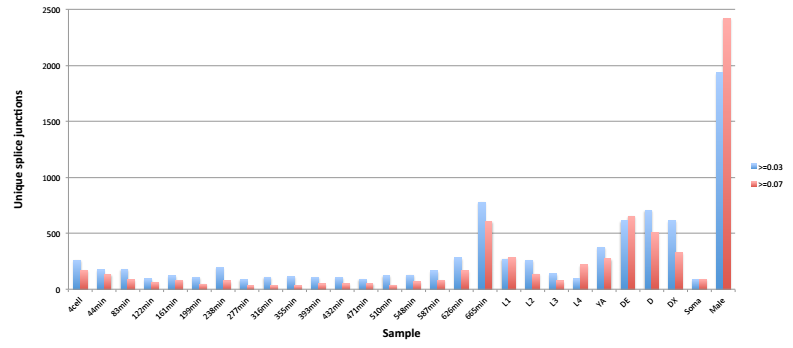


Supplemental Figure10. Unique introns per sample. The number of splice junctions unique to each sample at a dcpm threshold of 0.03 and 0.07 are shown.
