# Supplementary material for: The time-resolved transcriptome of C. elegans
Source: Genome Res. 2016 Oct;26(10):1441–50. doi: 10.1101/gr.202663.115 (PMC5052054; doi:10.1101/gr.202663.115)

Supplemental Figure 11. Representation of splice junctions overlapping and outside WormBase gene models. Splice junctions were divided into those that overlapped WormBase protein coding gene models and those that did not. In turn each junction was catalogued either as being unique or as having multiple partner junctions (alternatively spliced). A) The junctions binned by expression levels for junctions that have multiple partners. The distribution is clearly bimodal, with the peak on the left presumably derived from the minor isoform. B) Unique junctions, reflecting the range of expression of the associated genes. C, D) Novel junctions, unique or alternatively spliced. These junctions have much lower levels of expression. The absence of a second peak in the sites with multiple partners probably results from the low level of even the major isoform.


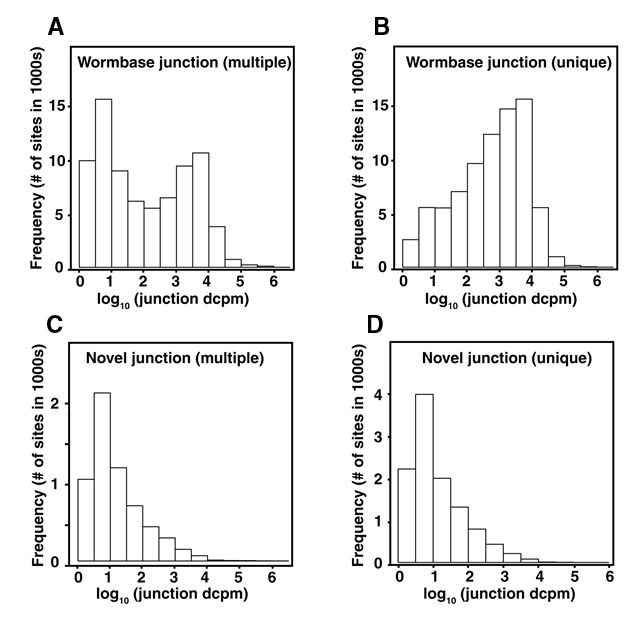

Supplement: Supplemental Material [file supp_gr.202663.115_Supplemental_Fig_S11.docx]
