# Supplementary material for: The time-resolved transcriptome of C. elegans
Source: Genome Res. 2016 Oct;26(10):1441–50. doi: 10.1101/gr.202663.115 (PMC5052054; doi:10.1101/gr.202663.115)

Supplemental Figure 12. Ratio of minor to major isoforms. A) The ratio of the number of reads spanning the minor isoform relative to major form is plotted against the log_10_ of the expression level of the minor isoform. Junctions where the minor form is annotated in WormBase are shown in black; those where the minor isoform is not present in WormBase are shown in red. B) An enlargement of the lower left region of A). In most pairs, the minor form is less than 5% of the major form and the number of reads representing the minor isoform is less than 100 reads in the aggregate data set. The presence of many WormBase annotated minor forms with a representation of less than 1% and an abundance of the minor form of less than 30 reads is notable.


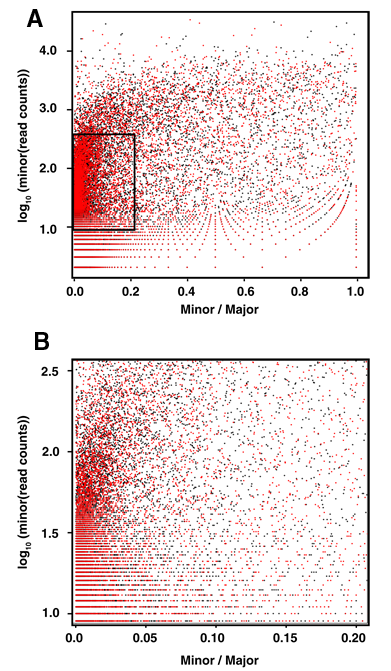

Supplement: Supplemental Material [file supp_gr.202663.115_Supplemental_Fig_S12.docx]
