# Supplementary material for: The time-resolved transcriptome of C. elegans
Source: Genome Res. 2016 Oct;26(10):1441–50. doi: 10.1101/gr.202663.115 (PMC5052054; doi:10.1101/gr.202663.115)
Supplement: Supplemental Material [file supp_gr.202663.115_Supplemental_Fig_S13.docx]

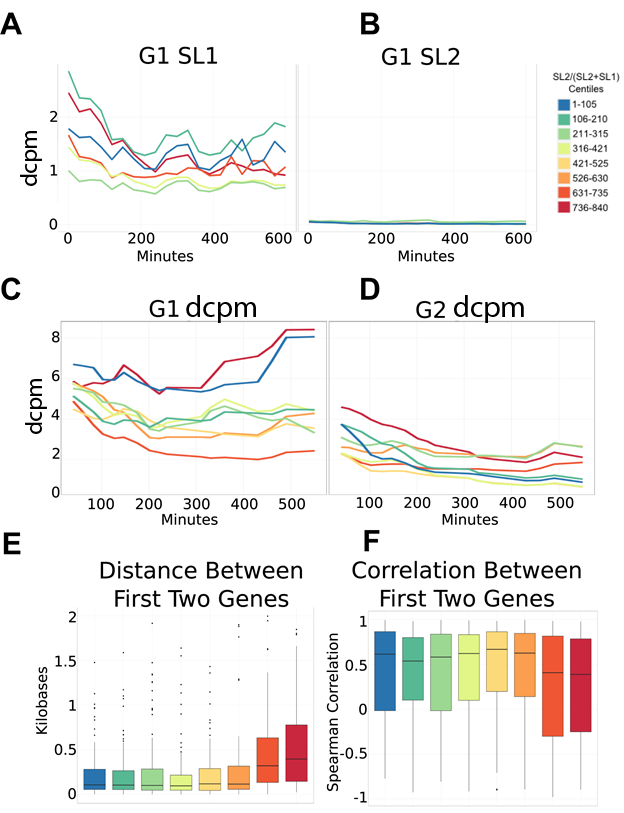


Supplemental Figure 13. Operon bin dynamics. Operons were binned according to the ratio of SL2/(SL2+SL1) at the second gene (G2) in the operon (see methods). All plots show 105-gene averages as in figure 3. Bins were colored as in figure 3. Minutes are shown post 2-cell division. A) Average SL1 dcpm during development for the first gene (G1) in an operon. There were no significant differences between any of the bins. B) Average SL2 dcpm during development for G1. Expression was below background for all time points and bins C) Average dcpm during development for G1. There was a slightly significant difference between the first and last bins when compared to the remaining 6 bins. D) Average dcpm during development for G2. There were no significant differences in any of the bins. E) Average distance in kilobases between the poly-A signal of G1 and the TSS of G2. Those bins with the highest SL2 ratio had the longest distance between the G1 polyA signal and the G2 TSS F) Spearman correlation between G1 and G2. Those bins with the highest SL2 ratio had a significantly lower correlation between G1 and G2.
