# Supplementary material for: The time-resolved transcriptome of C. elegans
Source: Genome Res. 2016 Oct;26(10):1441–50. doi: 10.1101/gr.202663.115 (PMC5052054; doi:10.1101/gr.202663.115)
Supplement: Supplemental Material [file supp_gr.202663.115_Supplemental_Fig_S14.docx]

Supplemental Figure 14. Average ChIP-seq signal surrounding the transcript start site of the first (left) and second (right) gene in all operons. ChIP-seq signal was binned into eight quantiles according to the second gene (G2) SL2/(SL2+SL1) ratio; the bin with the highest ratio is the top row as in Figure 4. The x-axis labels are also as in Figure 4 (Transcript Starts in basepairs ranging from -500 to 0 to + 500). Signal is shown from blue (lowest) to white (medium) to red (highest).
