# Supplementary material for: The time-resolved transcriptome of C. elegans
Source: Genome Res. 2016 Oct;26(10):1441–50. doi: 10.1101/gr.202663.115 (PMC5052054; doi:10.1101/gr.202663.115)

Supplemental Figure 15. A) DNA sequence motifs in the intergenic sequence between divergently transcribed histone genes. After removing closely related intergenic sequences, thirteen sequences were submitted to <http://meme-suite.org/tools/meme> for motif finding, using default parameters with the exception that motif length was limited to 27. Motifs 1 (red) and 2 (blue) are very similar to those previously described {Roberts et al, 1989; Roberts et al., 1987}. B) Each intergenic region has multiple copies of the motifs, generally with motif 3 most promoter proximal, followed by motif 1 and then motif 2. The maternally expressed histones (*his-46*, *his-63* and *his-66*} have a similar representation of motifs to the zygotic histones.


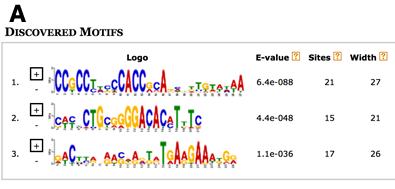

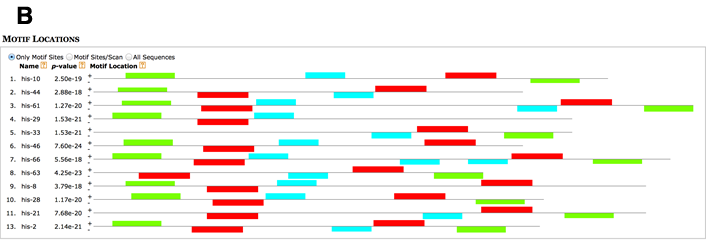

Supplement: Supplemental Material [file supp_gr.202663.115_Supplemental_Fig_S15.docx]
