# Supplementary material for: The time-resolved transcriptome of C. elegans
Source: Genome Res. 2016 Oct;26(10):1441–50. doi: 10.1101/gr.202663.115 (PMC5052054; doi:10.1101/gr.202663.115)

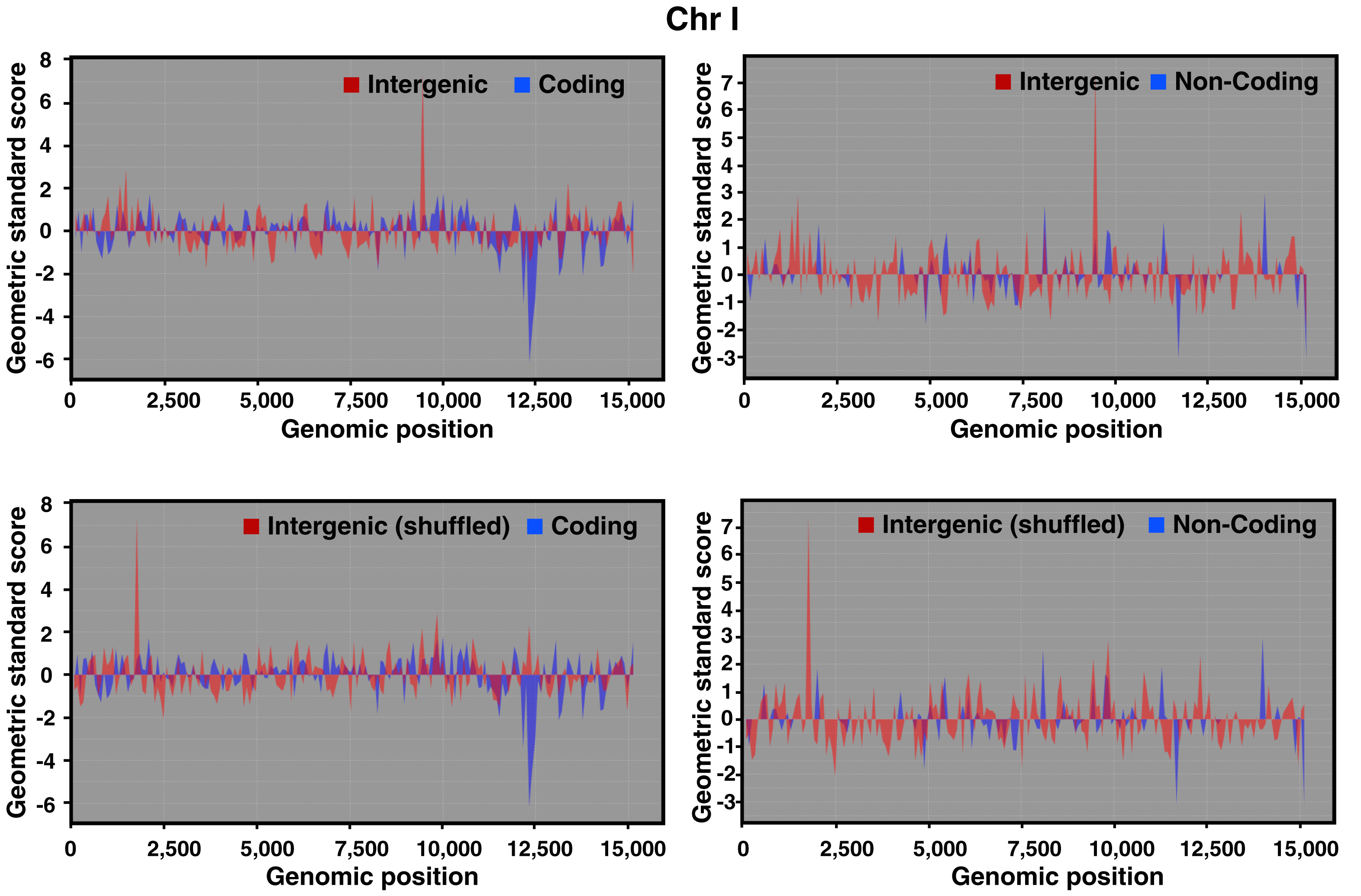

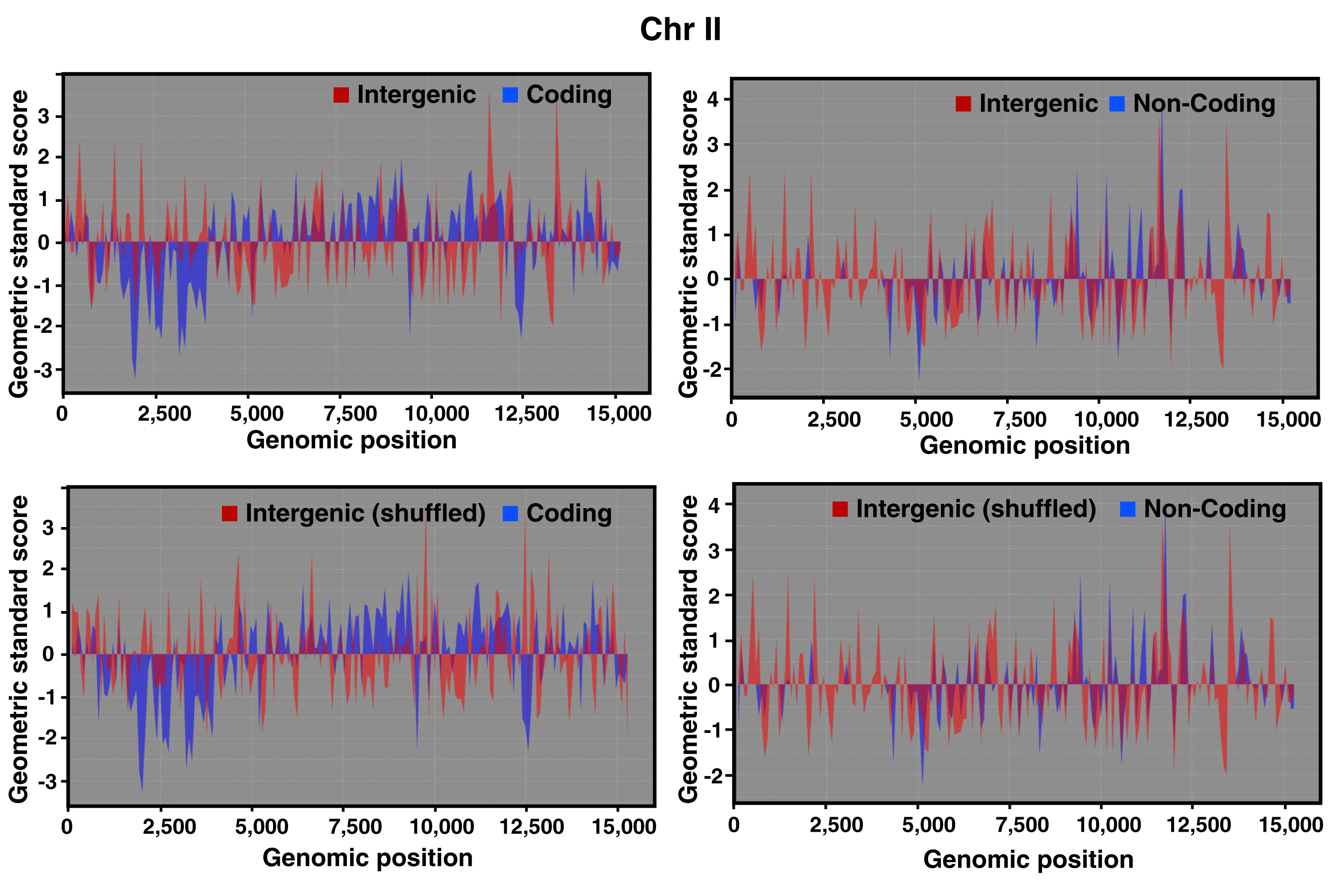


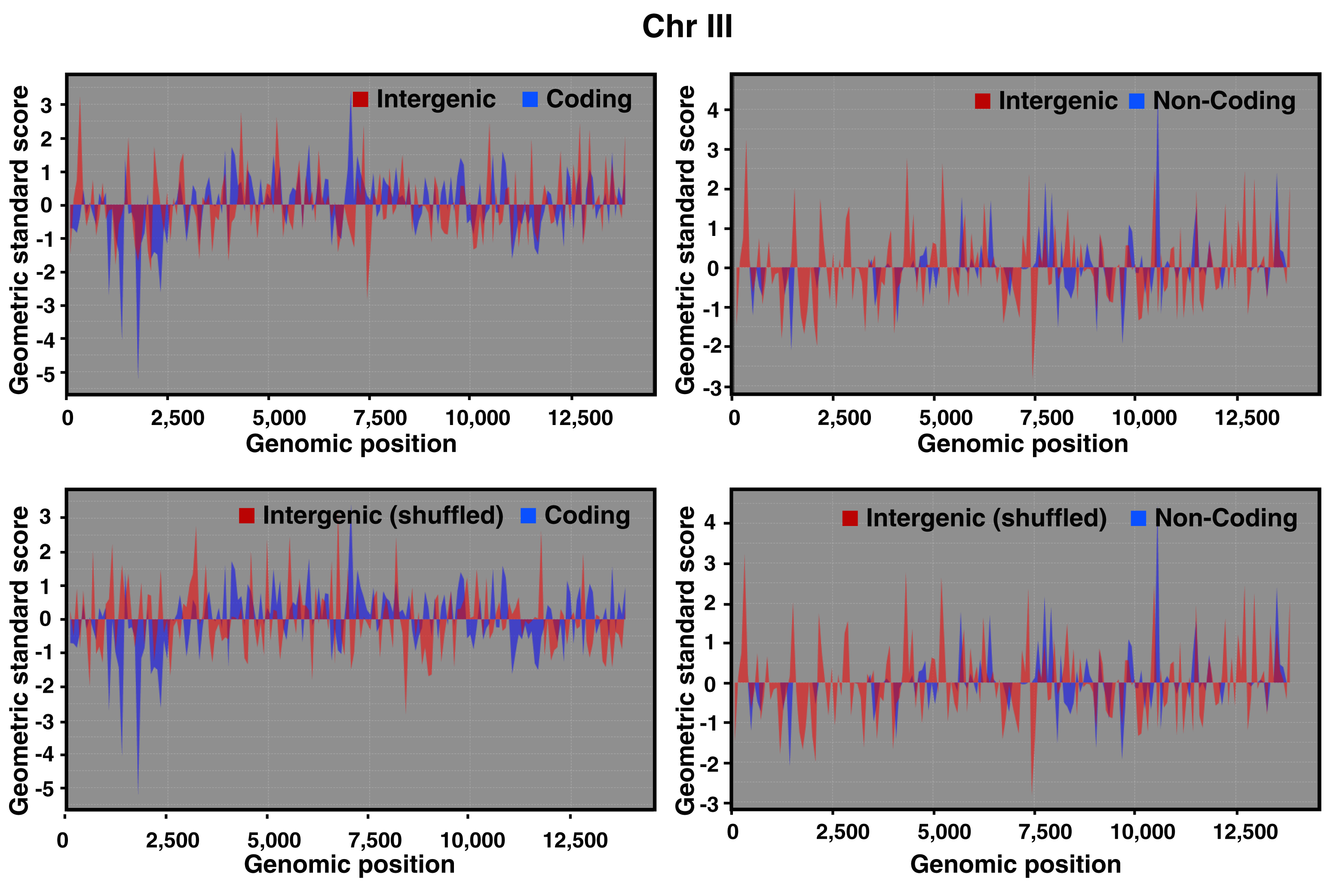


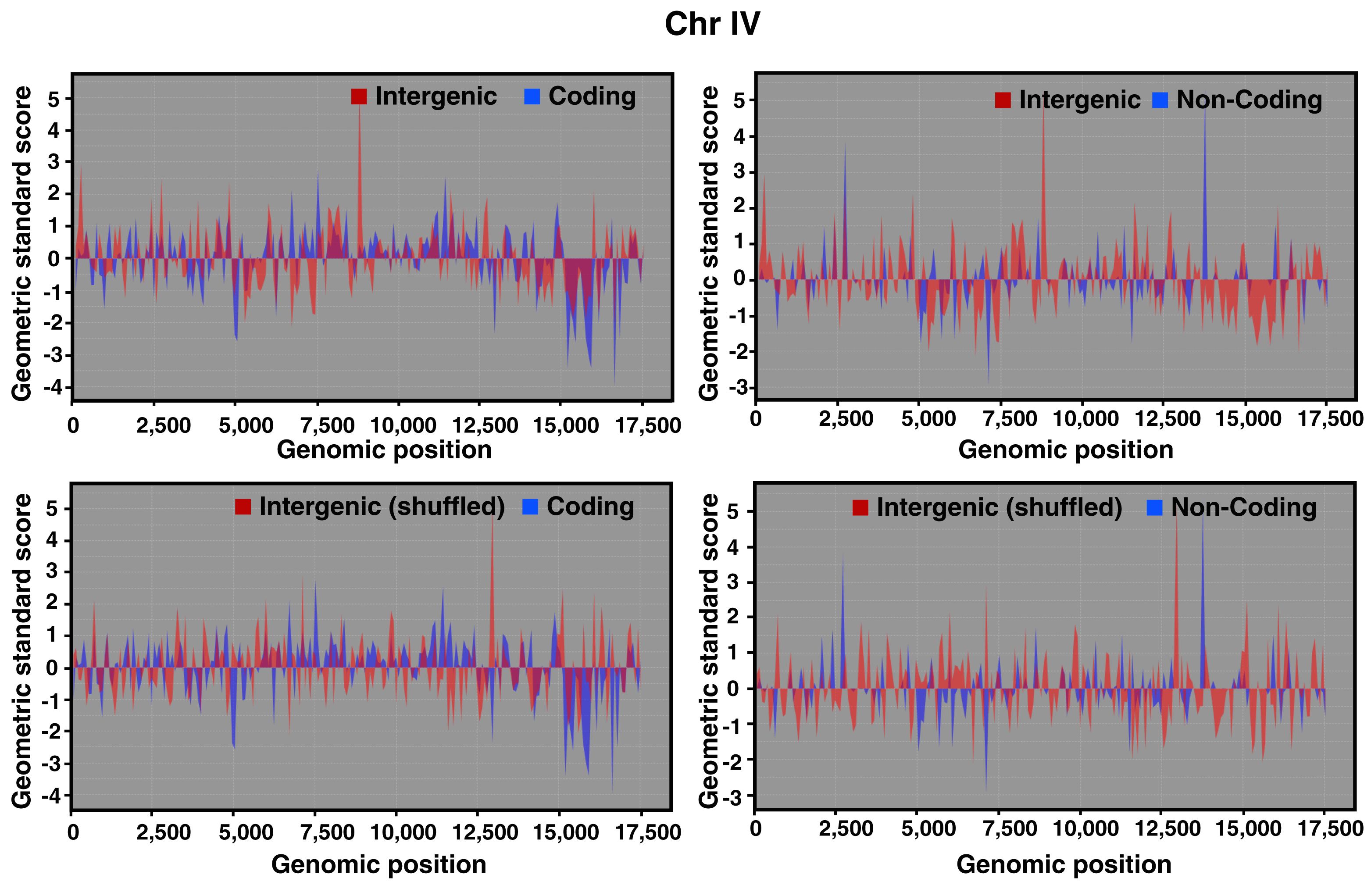


Supplemental Figure 16. Average dcpm values for 80kb regions for bases annotated as intergenic, protein coding gene, or non-coding gene in each 80kb region. The regional dcpm values were averaged across all high quality embryonic samples. The per-region dcpm values are normalized by transforming to the geometric standard score for each chromosome, to facilitate comparison of intergenic, coding and non-coding signals. The x-axis is the genomic position of the 80kb regions, in thousands of bases, for each chromosome. Charts with intergenic signal labeled shuffled show one example of a randomization of the regional intergenic values together with non-randomized coding and non-coding signals.


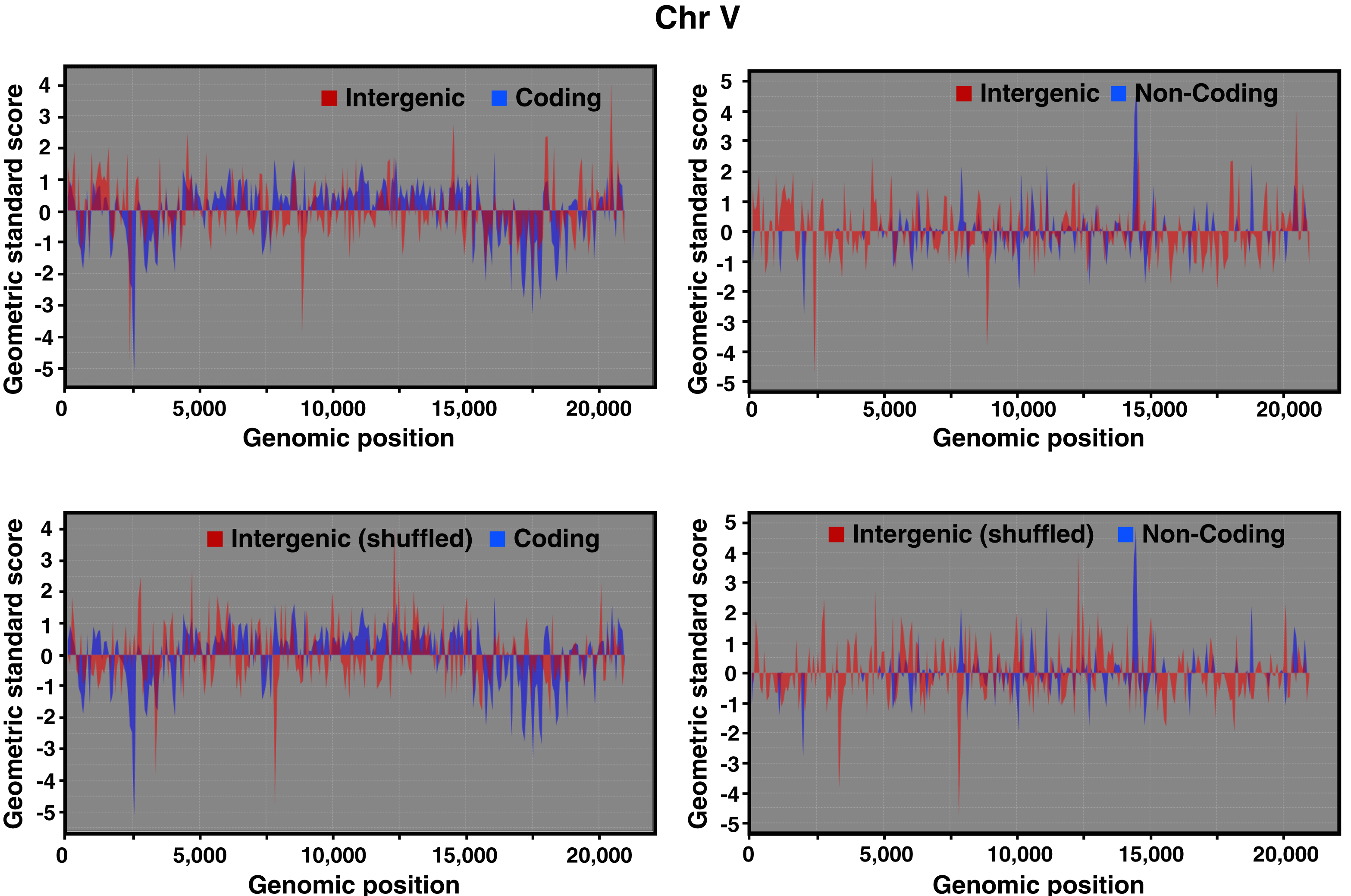


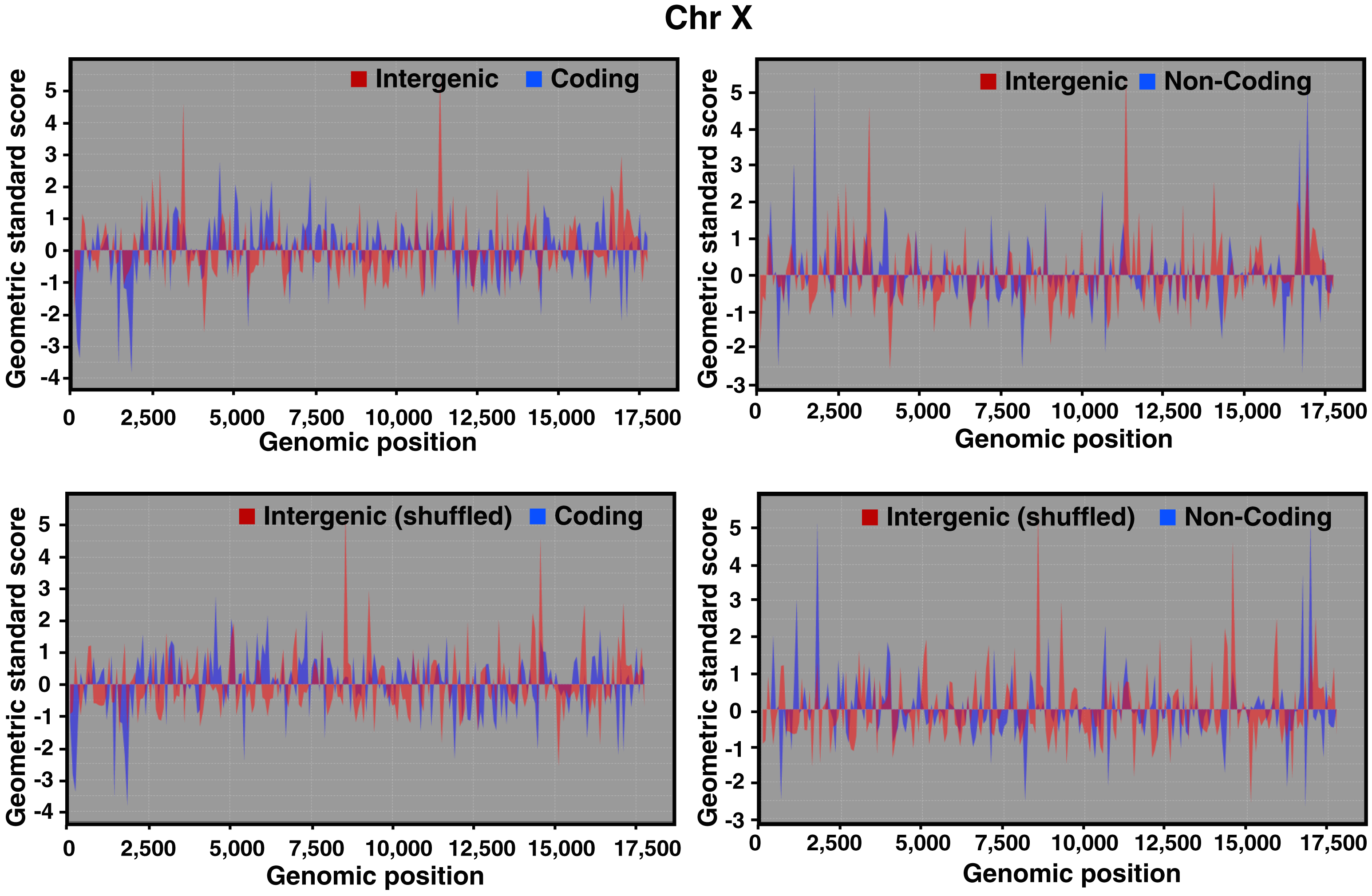

Supplement: Supplemental Material [file supp_gr.202663.115_Supplemental_Fig_S16.docx]
