# Supplementary material for: The time-resolved transcriptome of C. elegans
Source: Genome Res. 2016 Oct;26(10):1441–50. doi: 10.1101/gr.202663.115 (PMC5052054; doi:10.1101/gr.202663.115)
Supplement: Supplemental Material [file supp_gr.202663.115_Supplemental_Fig_S17.docx]

A) 5’ ends

Total RNA


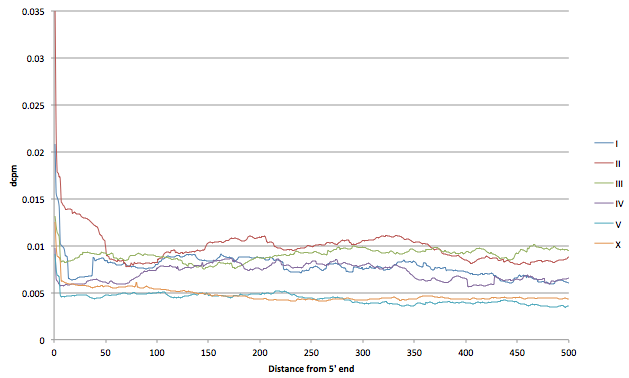


polyA+ RNA


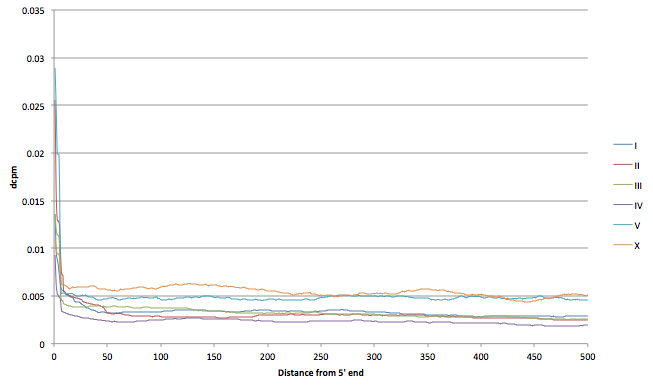


B) 3’ ends

Total RNA


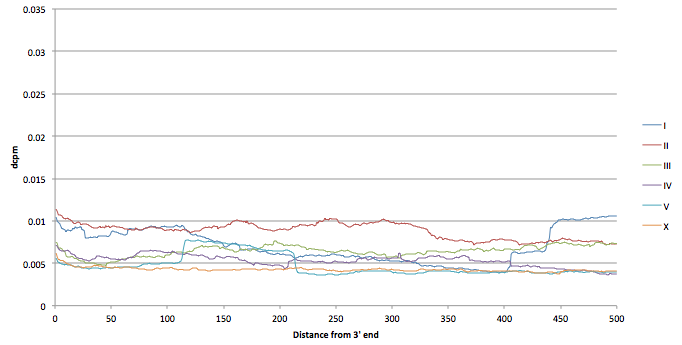


polyA+ RNA


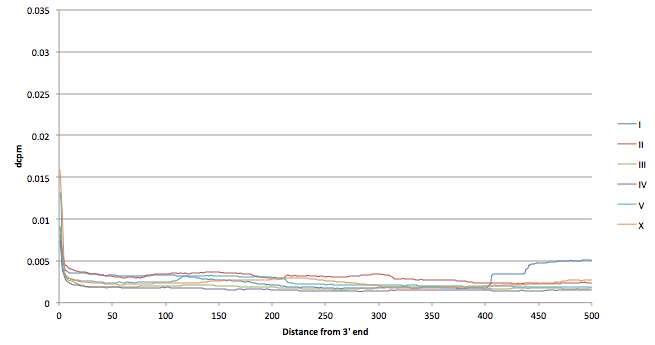


Supplemental Figure 17. Read coverage adjacent to the 5’ and 3’ edges of transcripts. Using the 5’ and 3’ ends of the aggregate transcript set, all adjacent regions were identified where there were at least 1000 bases not covered by any transcript (either an aggregate transcript or by any WormBase transcript). The aggregate read coverage across all extending for the adjacent 500 bases from the 5’ end was summed across all regions. Only samples with a 5% intergenic ROC threshold of <=200 were included (totalRNA: 20120223_EMB-0, 20120223_EMB-30, 20120223_EMB-60, 20120411_EMB-0, 20120411_EMB-120, 20120411_EMB-60, 20120419_EMB-120, 20120419_EMB-30; polyA+ N2_EE_50-0,N2_EE_50-210,N2_EE_50-240,N2_EE_50-30,N2_EE_50-330,N2_EE_50-360,N2_EE_50-390,N2_EE_50-420,N2_EE_50-450,N2_EE_50-480,N2_EE_50-510,N2_EE_50-540,N2_EE_50-570,N2_EE_50-60,N2_EE_50-600,N2_EE_50-630,N2_EE_50-660,N2_EE_50-690,N2_EE_50-720).
